# Mechanism of the Phospha-Wittig–Horner Reaction**

**DOI:** 10.1002/anie.201301469

**Published:** 2013-05-07

**Authors:** Anna I Arkhypchuk, Yurii V Svyaschenko, Andreas Orthaber, Sascha Ott

**Affiliations:** Department of Chemistry, Ångström Laboratories, Uppsala UniversityBox 523, 751 20 Uppsala (Sweden) E-mail: sascha.ott@kemi.uu.se

**Keywords:** ketenes, phosphaallenes, phospha-Wittig–Horner reaction, reaction mechanisms

Phosphaalkenes are prominent ligands in homogenous catalysis due to their π-acceptor capacity^1^ and are appealing building blocks in polymer science.1b, 2 Methods for their preparation are however often limited by small substrate scopes, or residual OTMS substituents that stem from certain synthetic methods.^3^ The phospha-Wittig–Horner (pWH) reaction, as first coined by Mathey and co-workers,^4^ converts aldehydes and relatively unreactive ketones into *C*-mono- and *C,C*-disubstituted phosphaalkenes, respectively (Scheme 1, top). Furthermore, the substituent at the phosphorus center can be chosen rather freely, although smaller groups demand additional stabilization by metal coordination.^5^ In context of our previous work on the incorporation of low-valent phosphorus into unsaturated carbon scaffolds,^6^ we were interested to see whether the scope of the pWH reaction could be extended to ketene substrates from which 1-phosphapropadienes would become accessible. Such phosphaallenes are the shortest members of an intriguing class of P-terminated cumulenes, which have however been synthetically highly challenging with only sporadic reports in the literature.^7^

**Scheme 1:**
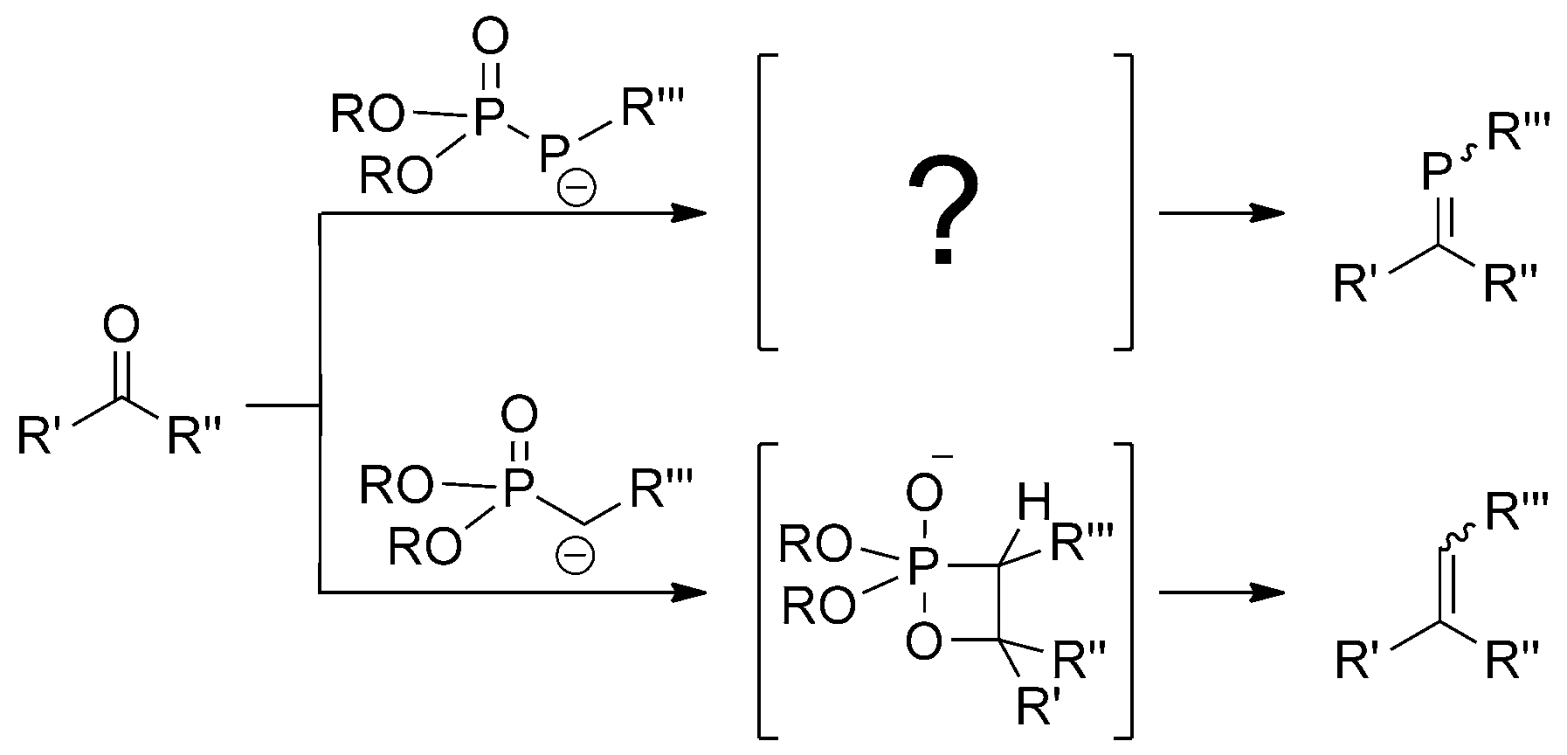
The phospha-Wittig–Horner reaction (top), for which the mechanism is unknown, and the carbon-analogue Horner–Wadsworth–Emmons reaction (bottom).

Despite of the synthetic versatility of the pWH reaction, it has been a greatly underexplored method for the preparation of phosphaalkenes. At the same time, the reaction mechanism has hitherto been unknown. Formally, the pWH reaction is the phosphorus analogue to the Horner–Wadsworth–Emmons reaction (HWE), which allows preparation of alkenes and allenes from carbonyl compounds and ketenes, respectively.^8^ The mechanism of the HWE reaction has been studied in great detail over the last decades, with the crucial step being a simultaneous C–P and C–O bond cleavage in an intermediately formed oxaphosphetane to afford the desired alkene and the phosphate byproduct (Scheme 1, bottom).^9^ In the spirit of the frequently drawn analogy between phosphorus and carbon,^10^ it was assumed that the pWH reaction proceeds along a similar pathway. In the present report, we show for the first time that this assumption is wrong and that the pWH reaction proceeds in a more complex, stepwise fashion. The mechanism of the pWH reaction is elucidated with the aid of spectroscopically and crystallographically characterized reaction intermediates.

The present study is based on two pWH reagents that are stabilized by {W(CO)_5_} fragments but differ in the P^III^ substituent (**1, 2**),5a and two substrate ketenes, one with phenyl substituents (**3**), the other with a fused fluorenyl core (**4**). As for all HWE and pWH reactions, the reactions are initiated by the addition of base. Thus, stoichiometric amounts of organic base (1,8-diazabicyclo[5.4.0]undec-7-ene, DBU) were added to **1** or **2** followed by the addition of either ketene **3** or **4**. The products that were obtained after 30 min and aqueous workup were however not the expected phosphaallenes, but previously unknown phosphinophosphates **5 a**–**d** (Scheme 2).

**Scheme 2:**
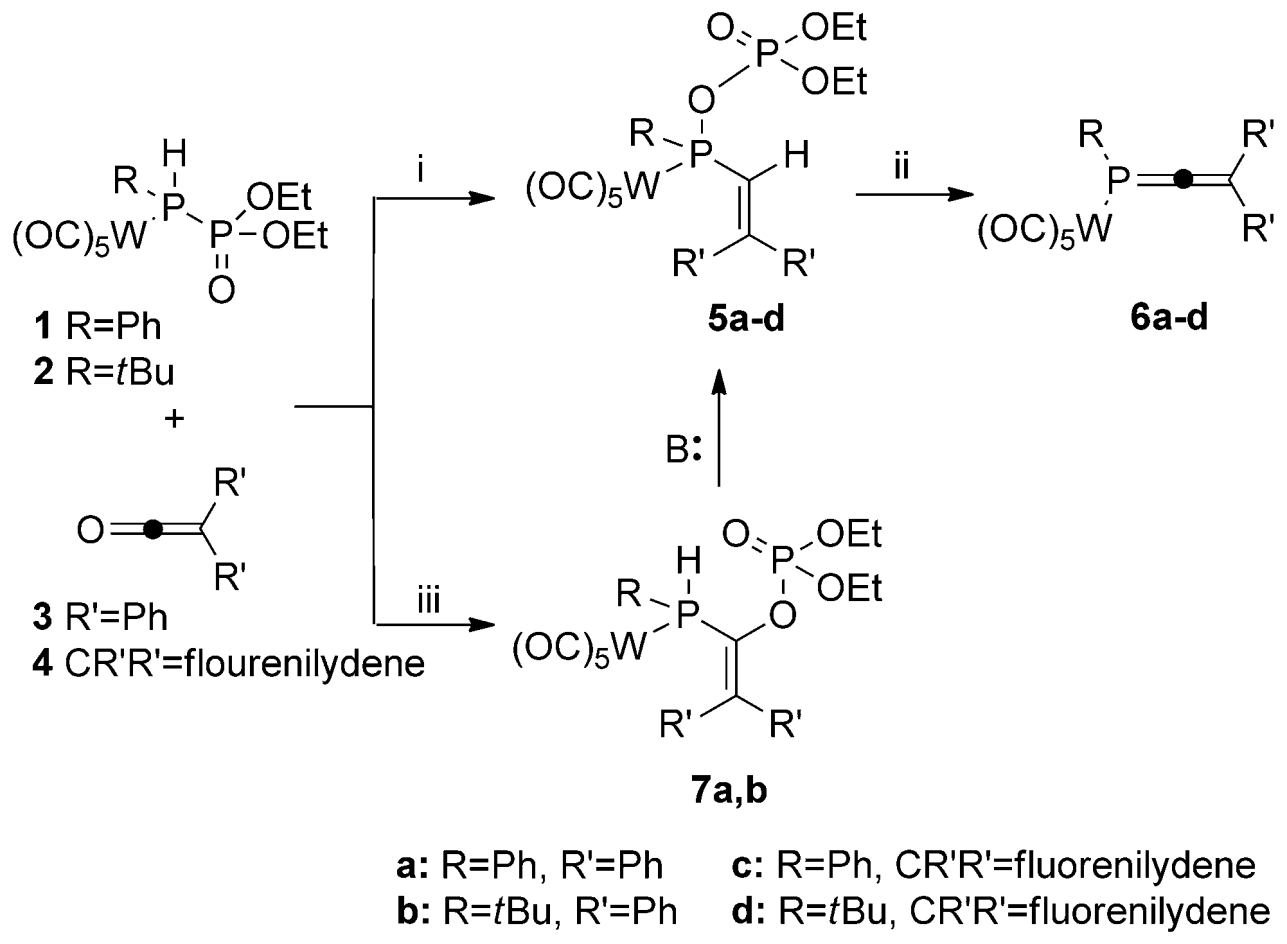
Reaction between **1**,**2** and **3**,**4** using DBU as base. Reagents and conditions: i) For ketene **3**: 1 equiv DBU, THF, RT, 30 min; ketene **4** was generated in situ from the corresponding acid chloride: 2 equiv of DBU, THF, RT. ii) 1 equiv DBU, THF, RT, 2–5 days. iii) 1) catalytic amounts of DBU, 5 min, 2) H_2_O.

Complexes **5 a**–**d** are obtained as pale yellow solids in good to excellent yields. They feature characteristic ^31^P NMR chemical shifts of −9.8 ppm for the P^V^, and 127 ppm (R=Ph, **5 a**,**c**) or 148 ppm (R=*t*Bu, **5 b,d**) for the P^III^ center. All of the NMR studies as well as HRMS data of **5 a**–**d** are in agreement with their proposed structures. Unambiguous evidence for the structure of **5** was obtained by single-crystal X-ray diffraction of compound **5 c**. As shown in Figure 1, the P^III^ exhibits a tetrahedral coordination sphere with a P1–C1 single bond (1.793(8) Å) and a C1–C2 double bond (1.344(10) Å).

**Figure 1:**
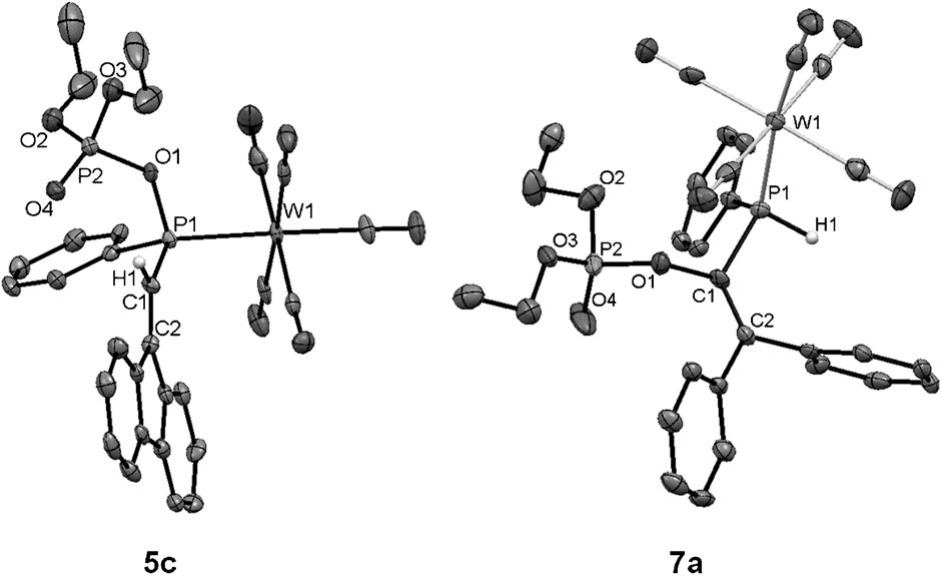
Crystal structures of compound **5 c** (left) and **7 a** (right). Ellipsoids set at 50 % probability; all hydrogen atoms are omitted for clarity, except for that at the vinyl moiety of **5 c**, and that of the P^III^ center in **7 a.** Only one of the disordered ethyl groups in **7 a** is displayed.

Extending the times for the reaction between **1**,**2** and **3**,**4** from 30 min to a couple of days leads to the formation of the desired phosphaallenes **6 a**–**d** (Scheme 2). Changing from an organic to an organolithium base (lithium diisopropylamide (LDA) or *t*BuOLi) accelerates the reaction significantly, and phosphaallenes **6 a**–**d** are obtained within one hour without detection of any reaction intermediates. Phosphaallenes **6 a**–**d** are characterized by typical ^31^P NMR chemical shifts of 57 ppm (**6 a**), 98 ppm (**6 b**), 60 ppm (**6 c**), and 94 ppm (**6 d**),7c are however highly moisture sensitive which hampers their isolation and purification. In the absence of extensive kinetic stabilization by large substituents at phosphorus, such behavior is not uncommon.7a Water (**8**) and methanol (**9**) addition products of **6** could however be isolated and characterized, in case of **8 b** and **8 c** even by single-crystal X-ray studies (Scheme 3 and Supporting Information).

**Scheme 3:**
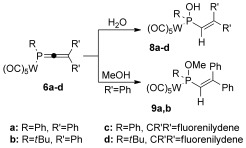
Isolated products from the solvolysis of phosphaallenes **6 a**–**d**.

Isolated compound **5** was re-exposed to the basic reaction conditions to confirm that **5**, or a deprotonated form of **5**, is a genuine intermediate in the pWH reaction, and not just a decomposition product. Indeed, exposure of **5 a**–**d** to DBU results in its conversion into the corresponding phosphaallenes **6 a**–**d** on similar timescales as those required in the original one-pot reaction (Scheme 2). It thus seems that the elimination of the phosphate group with the concomitant formation of the phosphaallene is the rate-limiting step of the sequence. Considering that the pWH reaction is relatively fast for simple ketones, such as acetone, and advances without any detectable reaction intermediates,5a it must be the presence of the C=C double bond in **5 a**–**d** that impedes the elimination step.

Further examination of the pWH reaction between **1** and **2** and ketene **3** allowed the isolation and characterization of another unprecedented reaction intermediate. When the reaction is conducted with sub-stoichiometric amounts of base, stable new products **7 a**,**b** can be detected under the reaction conditions (Scheme 2), and isolated and characterized by NMR spectroscopy and HRMS. Compounds **7 a**,**b** are addition products of **3** with **1** and **2**, respectively, with a phosphate and P^III^ group at the former internal ketene carbon.

The crystal structure of **7 a** (Figure 1) shows a distorted tetrahedral geometry around the phosphorus center P1 with similar P1–C1 and C1–C2 bond distances as in **5 c** (1.828(6) Å and 1.325(9) Å, respectively). Also, compound **7** can re-enter the pWH reaction simply by the addition of base. Its transformation to **5** occurs on a timescale of minutes, while the final elimination to afford **6** proceeds on a longer timescale, as described above.

The discovery of compounds **5** and **7** allows for a detailed mechanistic proposal for the pWH reaction that is different to that commonly believed for the HWE reaction (Scheme 1). The reaction starts with the formation of the salts of pWH reagents **1** and **2** which have partial P=P double bond character but also bear significant negative charge on the P^III^ center.5a, 11 Attack of the P^III^ nucleophile at the ketene leads to the formation of intermediate **A** (Scheme 4). In analogy to the HWE reaction and the formation of oxaphosphetane intermediates, subsequent intramolecular nulceophilic ring closure occurs also on **A**, and the corresponding oxadiphosphetane **B** is obtained. In contrast to what is apparent for the HWE reactions, however, intermediate **B** does not undergo a single-step phosphate elimination, but exclusive cleavage of the P–P single bond. The thus-formed intermediate **C** contains the newly formed phosphate group and bears negative charge on the P^III^ center. In case the reaction is performed with sub-stoichiometric amounts of base, intermediate **C** acts as a base and will deprotonate residual **1** or **2**. Supporting this notion, it was found in a separate experiment that **1** is a stronger acid than **7**, as the former exchanges its proton in anhydrous [D_4_]MeOH, while that of the latter remains unchanged. In the presence of sufficient amount of base, intermediate **C** remains in its anionic state und undergoes a [2,3]-sigmatropic rearrangement that leads to the final intermediate **D**. E2 elimination of the phosphate gives rise to the desired phosphaallenes **6 a**–**d**.

**Scheme 4:**
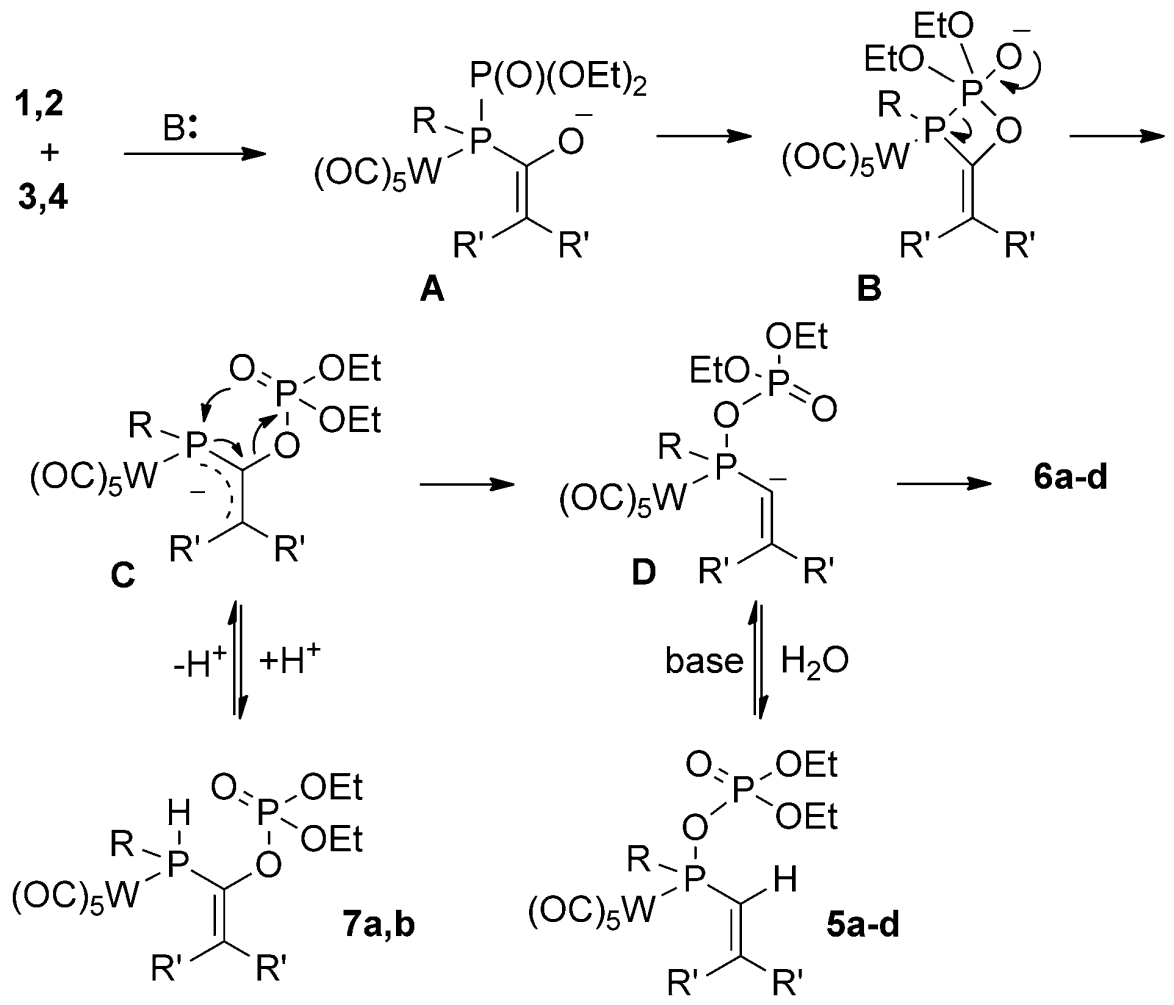
Proposed mechanism of the pWH reaction.

As indicated above, products of exclusive P–C bond cleavage of the oxaphosphetane intermediate in the HWE reaction have never been observed, and intermediates of type **5** and **7** are encountered only in the pWH reaction. Comparing the two reactions, it can be noted that the P–P bond in **B** is weaker than the corresponding C–P bond in oxaphosphetanes and can therefore be expected to be cleaved more easily. At the same time, the sp^2^-C–O bond in **B** is stronger than the sp^3^-C–O bond in the oxaphosphetane in Scheme 1. With **B** thus possessing a relatively weak P–P bond and a stronger C–O bond, it is not surprising any longer that selective cleavage of the P–P bond is observed. The negative charge in the resulting intermediate **C** is delocalized and thus best described as an allyl anion. The [2,3]-sigmatropic rearrangement to form intermediate **D** is the most puzzling finding in the reaction mechanism. Related [3,3] rearrangements have however precedence in the isomerization of allylic phosphates.^12^ The driving force for the formation of **D** is the creation of a new P–O bond and the formation of a relatively stable vinyl carbanion. Furthermore, intermediate **D** is kinetically stabilized as the final E2 elimination to form **6** requires an s-*trans* arrangement of the vinyl lone pair and the phosphate leaving group. Such a conformation demands the co-planarity of the entire POPC=C portion of the molecule which inevitably leads to severe steric clashes between the phosphate and the R’ groups. The steric argument is also reflected in the long reaction times that are observed for the transformation of **D** to **6 a**–**d**.

In summary, we have investigated the reactivity of pWH reagents **1** and **2** towards ketenes and were able to establish a reliable route to 1-phosphaallenes. The use of ketene substrates allowed the identification of unique reaction intermediates **7 a**,**b** as well as **5 a**–**d**, which offer a detailed mechanistic picture of the pWH reaction for the first time. Exclusive P–P bond cleavage of the oxadiphosphetane, followed by a [2,3]-sigmatropic rearrangement, establishes the phosphate leaving group at the P^III^. Subsequent E2 elimination is rate-limiting and establishes the 1-phosphaallenes. We believe that the increased mechanistic understanding provided herein will revive the pWH reaction and popularize its use for the preparation of phosphaalkenes in catalysis and materials science.

## Experimental Section

General procedure for compounds **5**: A solution of **1** or **2** in THF (1 mL) was added to a solution of 1 equiv of ketene **3** or **4** in THF (5 mL) at 20 °C. 1 equiv of of DBU in THF (1 m) was added dropwise, and the resulting reaction mixture was stirred for 5–30 min, during which time it was monitored by ^31^P NMR spectroscopy. Aqueous work-up was followed by purification by silica gel column chromatography using diethyl ether as eluent. **5 c**: 66 % yield of isolated product (see the Supporting Information for details). ^31^P NMR (CDCl_3_): *δ*=127.2 (d, ^1^*J*_PP_=49 Hz, ^1^*J*_PW_=293 Hz, P^III^), −9.9 ppm (d, P^V^). ^1^H NMR (CDCl_3_): *δ*=8.1–7.93 (m, 2 H, Ph), 7.83 (d, *J*=7 Hz, 1 H, Ar), 7.63 (d, *J*=7 Hz, 1 H, Ar), 7.59 (d, *J*=7.6 Hz, 1 H, Ar), 7.54–7.46 (m, 4 H, Ph, *H*C=), 7.39 (t, ^3^*J*_HH_=7 Hz, 1 H, Ar), 7.35 (t, ^3^*J*_HH_=7 Hz, 1 H, Ar), 7.28–7.20 (m, 1 H, Ar), 6.91–9.83 (m, 2 H, Ar), 4.15–3.93 (m, 2 H, OC*H*_2_CH_3_), 3.87–3.59 (m, 2 H, OC*H*_2_CH_3_), 1.28 (t, ^3^*J*_HH_=7 Hz, 3 H, OCH_2_C*H*_3_), 1.05 ppm (t, ^3^*J*_HH_=7 Hz, 3 H, OCH_2_C*H*_3_). ^13^C NMR (CDCl_3_): *δ*=198.4 (d, *J*=28 Hz), 195.8 (d, *J*_CW_=126 Hz, *J*_CP_=8 Hz), 146.7 (d, *J*=6 Hz), 142.2 (s), 140.4 (d, *J*=1 Hz), 138.2 (d, *J*=13.5 Hz), 134.7 (d, *J*=3 Hz), 134.6 (d, *J*=37 Hz), 132.9 (d, *J*=2 Hz), 132.6 (d, *J*=16 Hz), 130.1 (d, *J*=25 Hz), 129 (d, *J*=11 Hz), 129 (d, *J*=2 Hz), 127.7 (s), 126.5 (s), 123 (dd, *J*=40 Hz, *J*=3 Hz), 121.7 (s), 120 (s), 119.9 (s), 64.4 (d, *J*=6 Hz), 64.3 (d, *J*=6 Hz), 16. (d, *J*=7 Hz), 15.80 ppm (d, *J*=7 Hz). HRMS (solution in CHCl_3_ with addition of AgTFA): calcd for C_29_H_24_O_9_P_2_WAg, [*M*+Ag]^+^ 870.94683, found 870.94751.

General procedure for compounds **7**: A solution of **1** or **2** in THF (1 mL) was added to a solution of 1 equiv of ketene **3** or **4** in THF (5 mL) at 20 °C. One drop of a 1 m solution of DBU in THF was added. The reaction mixture was stirred for 5 min and then directly poured onto a silica gel column (short plug), and eluted with diethyl ether. Pure products were obtained by chromatography on silica gel with CH_2_Cl_2_ as eluent. **7 a**: 100 mg (0.176 mmol) of **1** and 36 mg (0.186 mmol) of ketene **3** was used for reaction. Yield: 130 mg; 97 %. ^31^P NMR (CD_2_Cl_2_): *δ*=−6.2 (d, P^V^), −26.3 ppm (d, ^1^*J*_PW_=232 Hz, P^III^). ^1^H NMR (dcm-d_2_): *δ*=7.94–7.66 (m, 2 H, Ph), 7.54–7.40 (m, 3 H, Ph), 7.39–7.21 (m, 10 H, Ph), 6.64 (d, ^1^*J*_HP_=365 Hz, 1 H, PH), 3.78–3.36 (m, 4 H, OC*H*_2_CH_3_), 1.09 (td, ^3^*J*_HH_=7 Hz, *J*_HP_=1 Hz, 3 H, OCH_2_C*H*_3_), 0.98 ppm (td, ^3^*J*_HH_=7 Hz, *J*_HP_=1 Hz, 3 H, OCH_2_C*H*_3_). ^13^C NMR (dcm-d_2_): *δ*=198.8 (d, *J*=23 Hz), 196.2 (d, *J*_CW_=126 Hz, *J*_CP_=7 Hz), 142.6 (dd, *J*=14 Hz, *J*=6 Hz), 141.3 (dd, *J*=57 Hz, *J*=9 Hz), 138.8 (dd, *J*=2 Hz, *J*=2 Hz), 138.2 (dd, *J*=5 Hz, *J*=2 Hz), 134.8 (d, *J*=13 Hz), 131.2 (d, *J*=2 Hz), 129.4 (d, *J*=1 Hz), 129 (s), 128.8 (d, *J*=10.6 Hz), 128.5 (s), 128.2 (s), 128.1 (s), 127.7 (d, *J*=42 Hz), 64.2 (d, *J*=6 Hz), 63.9 (d, *J*=6 Hz), 15.7 (d, *J*=7 Hz), 15.6 ppm (d, *J*=7.3 Hz). HRMS (solution in CHCl_3_/MeCN with addition of CF_3_COOAg): calcd for C_29_H_26_O_9_P_2_WAg, [*M*+Ag]^+^ 872.96248, found 872.96292.

## Supplementary material

As a service to our authors and readers, this journal provides supporting information supplied by the authors. Such materials are peer reviewed and may be re-organized for online delivery, but are not copy-edited or typeset. Technical support issues arising from supporting information (other than missing files) should be addressed to the authors.
