# Supplementary material for: Mechanism of the Phospha-Wittig–Horner Reaction
Source: Angew Chem Int Ed Engl. 2013 May 7;52(25):6484–7. doi: 10.1002/anie.201301469 (PMC3738942; doi:10.1002/anie.201301469)
Supplement: Supplementary file 1 [file anie0052-6484-SD1.pdf]

Angewandte Chemie
Eine Zeitschrift der Gesellschaft Deutscher Chemiker

Supporting Information
© Wiley-VCH 2013
69451 Weinheim, Germany

Mechanism of the Phospha-Wittig–Horner Reaction**

*Anna I. Arkhypchuk, Yurii V. Svyaschenko, Andreas Orthaber, and Sascha Ott**

anie_201301469_sm_miscellaneous_information.pdf

Table of content

General experimental section 4
Preparation of ketenes. 5
Preparation of compounds 5 5
Preparation of phosphaallenes 6 7
Preparation of compounds 7. 8
Preparation of compounds 8 and 9. 9
X-ray data for compounds 5c, 7a, 8b and 8c 12
Table SI1. Crystallographic data for complex 5c, 7a, 8b and 8c. 13
Summary of selected bond lengths and angles of compound 5c. 14
Summary of selected bond lengths and angles of compound 7a. 15
Summary of selected bond lengths and angles of compound 8b. 16
Summary of selected bond lengths and angles of compound 8c. 17
NMR data for complex 5a (^{1}H, ^{31}P, ^{13}C) 18
NMR data for complex 5b (^{1}H, ^{31}P, ^{13}C) 20
NMR data for complex 5c (^{1}H, ^{31}P, ^{13}C) 21
NMR data for complex 5d (^{1}H, ^{31}P, ^{13}C) 23
NMR data for complex 6a (^{31}P) 24
NMR data for complex 6b (^{31}P) 25
NMR data for complex 6c (^{31}P) 26
NMR data for complex 6d (^{31}P) 27
NMR data for complex 7a (^{1}H, ^{31}P, ^{13}C) 28
NMR data for complex 7b (^{1}H, ^{31}P, ^{13}C) 29
NMR data for complex 8a (^{1}H, ^{31}P, ^{13}C) 31
NMR data for complex 8b (^{1}H, ^{31}P, ^{13}C) 32
NMR data for complex 8c (^{1}H, ^{31}P, ^{13}C) 34
NMR data for complex 8d (^{1}H, ^{31}P, ^{13}C) 35
NMR data for complex 9a (^{1}H, ^{31}P, ^{13}C) 37
NMR data for complex 9b (^{1}H, ^{31}P, ^{13}C) 38
References 40

General experimental section

General. All reactions were performed under argon using Schlenk techniques. Diethyl ether and THF were freshly distilled from sodium/benzophenone prior to use. ^{1}H, ^{13}C and ^{31}P spectra were recorded on a 400 MHz and 300 MHz spectrometers. Chemical shifts (ppm) were reported and referenced to the internal signal of residual protic solvent. High resolution mass spectral analyses (HRMS) were performed on high resolution and FTMS+pNSI mass spectrometer (OrbitrapXL).

X-ray data.

Crystallographic data sets were collected from single crystal samples mounted on a loop fiber and coated with N-paratone oil (Hampton Research). Collection was performed using a Bruker SMART APEX diffractometer equiped with an APEXII CCD detector, a graphite monochromator and a 3-circles goniometer. The crystal-to-detector distance was 5.0 cm, and the data collection was carried out in 512 x 512 pixel mode. The initial unit cell parameters were determined by a least-squares fit of the angular setting of strong reflections, collected by a 10.0 degrees scan in 33 frames over three different parts of the reciprocal space (99 frames total). Cell refinement and data reduction were performed with SAINT V7.68A (Bruker AXS). Absorption correction was done by multi-scan methods using SADABS96 (Sheldrick). The structure was solved by direct methods and refined using SHELXL97 (Sheldrick). All non-H atoms were refined by full-matrix least-squares with anisotropic displacement parameters while hydrogen atoms were placed in idealized positions. Refinement of F2 was performed against all reflections. The weighted R-factor wR and goodness of fit S are based on F2. Full details concerning the data sets and crystal resolutions can be found in the respective CIF files deposited at the Cambridge Crystallographic Data Centre under the allocated deposition numbers CCDC 924983 (**5c**), CCDC 924984 (**7a**), CCDC 924985 (**8b**) and CCDC 924986 (**8c**).

Preparation of ketenes.

Diphenyl ketene 3. Compound **3** was prepared by literature procedures and distilled prior of use.[1]

Flourenilyden ketene 4.

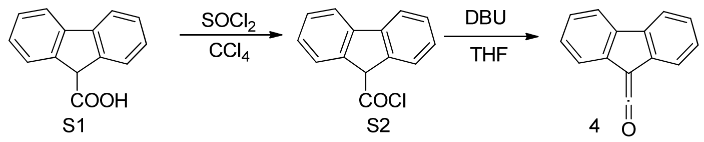

Scheme ESI 1. Preparation of 4.

Compound **4** was generated *in situ* by treatment of 1 eq. of the corresponding acid chloride **S2** with 1 eq. of DBU in 10-15ml of THF for 30min. Resulted dark red suspension was used directly in next step. Compound **S2** was prepared according to literature procedure. [2] Commercially available thionyl chloride was distilled under argon with olive oil prior to use.

Preparation of compounds 5

General procedure. To the solution of 1eq. of ketene **3** or **4** in 5ml of THF at r.t. solution of 1 eq. of corresponding pWHr was added at once. To the resulted mixture 1 eq. of 1M solution of DBU in THF was added drop wise. Resulted reaction mixture was stirred for 5-30 minutes (reaction was monitored by ^{31}P NMR) and quenched with water. Extraction with diethyl ether (3×30ml), drying with brine and $MgSO_4$ gave oily crude products **5**. Clean compounds were obtained by column chromatography on silica gel in diethyl ether.

Complex 5a. 100mg (0.176mmol) of 1 and 34mg (0.176mmol) of **3** and 0.176ml (0.176mmol) of 1M solution of DBU was used for reaction. Yield: 60mg; 45%.

^{31}P NMR ($CDCl_3$): δ = 125.0 (d, $^1J_{PP}$=41 Hz, $^1J_{PW}$=295 Hz, ^{III}P), -9.8 (d, ^{V}P) ppm. ^{1}H NMR ($CDCl_3$): δ = 7.72-7.67 (m, 2H, Ph), 7.32-7.26 (m, 8H, Ph), 7.07-7.00 (m, 3H, Ph), 6.96-6.97 (m, 2H, Ph), 6.91 (dm, $^2J_{HP}$=21 Hz, 1H, $\underline{H}$C=), 4.12-3.94 (m, 2H, $OC\underline{H}_2CH_3$), 3.87-3.73 (m, 2H, $OC\underline{H}_2CH_3$), 1.24 (tdd, J_{HP}=1 Hz, J_{HP}=2 Hz, $^3J_{HH}$=7 Hz, 3H, $OCH_2C\underline{H}_3$), 1.08 (tdd, J_{HP}=1 Hz, J_{HP}=2 Hz, $^3J_{HH}$=7 Hz, 3H, $OCH_2C\underline{H}_3$) ppm. ^{13}C NMR ($CDCl_3$): δ = 198.7 (d, J=28Hz), 195.9 (d, J_{CP}=8 Hz, J_{CW}=126 Hz), 154.0 (d, J=2 Hz), 141.9 (d, J=12 Hz), 137.6 (d, J=4 Hz), 135.9 (dd, J=40 Hz, J=2 Hz), 131.9 (s), 131.7 (s), 131.3 (s), 129.8 (s), 128.9 (s), 128.2 (d, J=4 Hz), 128.0 (s), 127.9 (d, J=11 Hz), 127.6 (s), 127.6 (dd, J=41 Hz, J=3 Hz), 64.0 (d, J=3 Hz),

63.9 (d, J=3 Hz), 15.7 (d, J=7 Hz), 15.5 (d, J=7 Hz) ppm. HRMS (solution in $CHCl_3$ with addition of AgTFA): calc. for $C_{29}H_{26}O_9P_2WAg$, $[M+Ag]^+$ 872.96248, found 872.96317.

Complex 5b. 50mg (0.094mmol) of **2** and 18mg (0.094mmol) of **3** and 0.094ml (0.094mmol) of 1M solution of DBU was used for reaction. Yield: 26mg; 37%.

^{31}P NMR (dcm-d$_2$): δ = 148.2 (d, $^1J_{PP}$=20 Hz, $^1J_{PW}$=279 Hz, ^{III}P), -9.7 (d, ^{V}P) ppm. ^1H NMR (dcm-d$_2$): δ = 7.35-7.32 (m, 3H, Ph), 7.31-7.27 (m, 3H, Ph), 7.27-7.23 (m, 2H, Ph), 7.19-7.16 (m, 2H, Ph), 6.25 (dd, $^2J_{HP}$=39 Hz, $^4J_{HP}$=5 Hz, 1H, $\underline{H}$C=), 3.86-3.65 (m, 4H, $OC\underline{H}_2CH_3$), 1.35 (d, $^3J_{HP}$=16 Hz, 9H, *t*Bu), 1.20 (dt, $^3J_{HH}$=7 Hz, $^3J_{HP}$=1 Hz, 3H, $OCH_2C\underline{H}_3$), 1.19 (dt, $^3J_{HH}$=7 Hz, $^3J_{HP}$=1 Hz, 3H, $OCH_2C\underline{H}_3$) ppm. ^{13}C NMR (dcm-d$_2$): δ = 198.6 (d, J=28 Hz), 196.6 (d, J_{CP}=7 Hz, J_{CW}=126 Hz), 152.9 (d, J=3 Hz), 143.4 (d, J=12 Hz), 138.4 (d, J=4 Hz), 129.6 (d, J=1 Hz), 128.7 (s), 128.4 (s), 128.1 (s), 127.7 (s), 127.7 (s), 124.2 (dd, J=35 Hz, J=6 Hz), 64.0 (d, J=6 Hz), 63.8 (d, J=6 Hz), 25.6 (d, J=7 Hz), 15.8 (d, J=8 Hz), 15.6 (d, J=8 Hz) ppm. HRMS (solution in $CHCl_3$ with addition of AgTFA): calc. for $C_{27}H_{30}O_9P_2WAg$, $[M+Ag]^+$ 852.99376, found 852.99472.

Complex 5c. 248mg (0.437mmol) of **1** and 0.437mmol of **4** (generated from 100mg (0.437mmol) of **S2** with 0.437ml of 1M solution in THF of DBU) and 0.437ml (0.437mmol) of 1M solution of DBU were used for reaction. Yield: 220mg; 66%.

^{31}P NMR ($CDCl_3$): δ = 127.2 (d, $^1J_{PP}$=49 Hz, $^1J_{PW}$=293 Hz, ^{III}P), -9.9 (d, ^{V}P) ppm. ^1H NMR ($CDCl_3$): δ = 8.1-7.93 (m, 2H, Ph), 7.83 (d, J=7Hz, 1H, Ar), 7.63 (d, J=7 Hz, 1H, Ar), 7.59 (d, J=7.6 Hz, 1H, Ar), 7.54-7.46 (m, 4H, Ph, $\underline{H}$C=), 7.39 (t, $^3J_{HH}$=7 Hz, 1H, Ar), 7.35 (t, $^3J_{HH}$=7 Hz, 1H, Ar), 7.28-7.20 (m, 1H, Ar), 6.91-9.83 (m, 2H, Ar), 4.15-3.93 (m, 2H, $OC\underline{H}_2CH_3$), 3.87-3.59 (m, 2H, $OC\underline{H}_2CH_3$), 1.28 (t, $^3J_{HH}$=7 Hz, 3H, $OCH_2C\underline{H}_3$), 1.05 (t, $^3J_{HH}$=7 Hz, 3H, $OCH_2C\underline{H}_3$) ppm. ^{13}C NMR ($CDCl_3$): δ = 198.4 (d, J=28 Hz), 195.8 (d, J_{CW}=126 Hz, J_{CP}=8 Hz), 146.7 (d, J=6 Hz), 142.2 (s), 140.4 (d, J=1 Hz), 138.2 (d, J=13.5 Hz), 134.7 (d, J=3 Hz), 134.6 (d, J=37 Hz), 132.9 (d, J=2 Hz), 132.6 (d, J=16 Hz), 130.1 (d, J=25 Hz), 129 (d, J=11 Hz), 129 (d, J=2 Hz), 127.7 (s), 126.5 (s), 123 (dd, J=40 Hz, J=3 Hz), 121.7 (s), 120 (s), 119.9 (s), 64.4 (d, J=6 Hz), 64.3 (d, J=6 Hz), 16. (d, J=7 Hz), 15.80 (d, J=7 Hz) ppm. HRMS (solution in $CHCl_3$ with addition of AgTFA): calc. for $C_{29}H_{24}O_9P_2WAg$, $[M+Ag]^+$ 870.94683, found 870.94751.

Complex 5d. 116mg (0.219mmol) of **2** and 0.219mmol of **4** (generated from 50mg (0.219mmol) of **S2** with 0.219ml of 1M solution in THF of DBU) and 0.219ml of 1M solution (0.219mmol) of DBU were used for reaction. Yield: 84mg; 51%.

^{31}P NMR (dcm-d_2): δ = 148.94 (d, J_{PW}=283Hz, $^1J_{PP}$=32 Hz, ^{III}P), -9.81 (d, ^{V}P) ppm. ^{1}H NMR (dcm-d_2): δ = 8.29 (d, $^3J_{HH}$=8 Hz, Ar), 7.73-7.60 (m, 3H, Ar), 7.46-7.34 (m, 2H, Ar), 7.34-7.25 (m, 2H, Ar), 6.94 (dd, $^2J_{HP}$=25 Hz, J_{HH}=4 Hz, 1H, $\underline{\text{H}}$C=), 4.19-4.11 (m, 2H, OC$\underline{\text{H}}$2CH3), 4.09-3.98 (m, 2H, OC$\underline{\text{H}}$2CH3), 1.46 (d, $^3J_{HP}$=16 Hz, 9H, *t*Bu), 1.33 (td, $^3J_{HH}$=7 Hz, $^4J_{HP}$=1 Hz, 3H, $OCH_2C\underline{H}_3$), 1.22 (td, $^3J_{HH}$=7 Hz, $^4J_{HP}$=1 Hz, 3H, $OCH_2C\underline{H}_3$) ppm. ^{13}C NMR (dcm-d_2): δ = 198.0 (d, *J*=28 Hz), 196.3 (d, *J*=7 Hz), 146.9 (d, *J*=2 Hz), 142.1 (s), 139.9 (s), 138.9 (d, *J*=12 Hz), 135.0 (d, *J*=3 Hz), 130.2 (s), 130.0 (s), 129.1 (s), 127.5 (s), 127.0 (s), 121.0 (dd, *J*=33 Hz, *J*=6 Hz), 120.7 (s), 119.8 (s), 119.8 (s), 64.8 (d, *J*=6 Hz), 64.7 (d, *J*=6 Hz), 26.0 (d, *J*=7 Hz), 15.8 (d, *J*=6 Hz), 15.7 (d, *J*=6 Hz) ppm. HRMS (solution in $CHCl_3$/ACN with addition of AgTFA): calc. for $C_{27}H_{28}O_9P_2WAg$, $[M+Ag]^+$ 850.97811, found 850.97833.

Preparation of phosphaallenes 6

Method A. To the mixture of 1 eq. of the ketene **3** or **4** and 1 eq. of the pWHr **1** or **2** in 10 ml THF, 2 eq. of 1M DBU solution in THF was added at room temperature. Reaction was followed by NMR. Complete conversion to the phosphaallenes **6a-d** was observed after 3-5days.

Method B. To the solution of the 1 eq. of pWHr **2** eq. of the 1M solution of tBuOLi in THF was added dropwise at -78°C. Reaction mixture stirred for 5 minutes and solution of the corresponding acid chloride **S2** in 2 ml THF was added. Reaction mixture allowed to warm up to r.t. After this complete conversion to the phosphaallene **6a-d** was observed.

Method C. To the solution of the 1 eq. of pWHr **1** or **2**, 1 eq. of the 1M solution of *t*BuOLi in THF was added dropwise at -78°C. Reaction mixture stirred for 5 minutes and solution of the corresponding ketene or **3** in 2 ml THF or solution of **4** prepared as described above was added. Reaction mixture allowed to warm up to r.t. After this complete conversion to the phosphaallene **6a-d** was observed.

Method D. To the solution of 1 eq. of complex 5a-d in THF 1 eq. of LDA (2M solution in THF/heptane/ethyl benzene) was added drop wise at -78°C. ^{31}P NMR after warming up to r.t. shows clean comversion to the corresponding phosphaallenes 5a-d.

Phosphaallene 6a. ^{31}P NMR (THF solution, C_6D_6 as internal standard): δ = 57.0 ($^1J_{PW}$=242 Hz) ppm.

Phosphaallene 6b. ^{31}P NMR (THF solution, C_6D_6 as internal standard): δ = 98.8 ($^1J_{PW}$=245 Hz) ppm.

Phosphaallene 6c. ^{31}P NMR (THF solution, C_6D_6 as internal standard): δ = 59.8 ($^{1}J_{PW}$=245 Hz) ppm.

Phosphaallene 6d. ^{31}P NMR (THF solution, C_6D_6 as internal standard): δ = 93.6 ($^{1}J_{PW}$=245 Hz) ppm.

Preparation of compounds 7.

General procedure. To the mixture of 1.05 eq. of ketene **3** and 1 eq. of pWHr **1** or **2** in 10ml of THF 1 drop of 1M solution of DBU in THF was added at r.t. Reaction mixture was stirred for 5 minutes and directly purred on silica gel column (short plug) and elueted with diethyl ether. Final product was obtained by chromatography on silica gel with DCM as eluent.

Compound 7a. 100mg (0.176mmol) of **1** and 36mg (0.186mmol) of ketene **3** was used for reaction. Yield: 130mg; 97%.

^{31}P NMR (dcm-d$_2$): δ = -6.2 (d, ^{V}P), -26.3 (d, $^{1}J_{PW}$=232 Hz, ^{III}P) ppm. ^{1}H NMR (dcm-d$_2$): δ = 7.94-7.66 (m, 2H, Ph), 7.54-7.40 (m, 3H, Ph), 7.39-7.21 (m, 10H, Ph), 6.64 (d, $^{1}J_{HP}$=365 Hz, 1H, PH), 3.78-3.36 (m, 4H, OC$\underline{H}_2$CH$_3$), 1.09 (td, $^{3}J_{HH}$= 7 Hz, J_{HP}=1 Hz, 3H, OCH$_2$C$\underline{H}_3$), 0.98 (td, $^{3}J_{HH}$=7 Hz, J_{HP}=1 Hz, 3H, OCH$_2$C$\underline{H}_3$) ppm. ^{13}C NMR (dcm-d$_2$): δ = 198.8 (d, *J*=23Hz), 196.2 (d, J_{CW}=126 Hz, J_{CP}=7 Hz), 142.6 (dd, *J*=14 Hz, *J*=6 Hz), 141.3 (dd, *J*=57 Hz, *J*=9 Hz), 138.8 (dd, *J*=2 Hz, *J*=2 Hz), 138.2 (dd, *J*=5 Hz, *J*=2 Hz), 134.8 (d, *J*=13 Hz), 131.2 (d, *J*=2 Hz), 129.4 (d, *J*=1 Hz), 129 (s), 128.8 (d, *J*=10.6 Hz), 128.5 (s), 128.2 (s), 128.1 (s), 127.7 (d, *J*=42 Hz), 64.2 (d, *J*=6 Hz), 63.9 (d, *J*=6 Hz), 15.7 (d, *J*=7 Hz), 15.6 (d, *J*=7.3 Hz) ppm. HRMS (solution in $CHCl_3$/ACN with addition of AgTFA): calc. for $C_{29}H_{26}O_9P_2WAg$, $[M+Ag]^+$ 872.96248, found 872.96292.

Compound 7b. 94mg (0.178mmol) of **2** and 36mg (0.186mmol) of ketene **3** was used for reaction. Yield: 90mg; 68%.

^{31}P NMR (dcm-d$_2$): δ = 0.63 (d, $^{1}J_{PP}$=7 Hz, $^{1}J_{PW}$=259 Hz, ^{III}P), -7.0 (d, ^{V}P) ppm. ^{1}H NMR (dcm-d$_2$): δ = 7.41-7.39 (m, 4H, Ph), 7.32-7.30 (m, 4H, Ph), 7.29-7.24 (m, 2H, Ph), 5.1 (d, $^{1}J_{HP}$=348 Hz, 1H, $\underline{H}$P), 3.92-3.52 (m, 4H, OC$\underline{H}_2$CH$_3$), 1.33 (d, $^{3}J_{HP}$=17 Hz, 9H, *t*Bu), 1.16 (dt, $^{3}J_{HH}$=7 Hz, $^{3}J_{HP}$=1 Hz, 3H, OCH$_2$C$\underline{H}_3$), 1.08 (dt, $^{3}J_{HH}$=7 Hz, $^{3}J_{HP}$=1 Hz, 3H, OCH$_2$C$\underline{H}_3$) ppm. ^{13}C NMR (dcm-d$_2$): δ = 198.5 (d, *J*=23 Hz), 196.6 (d, J_{CP}=7 Hz, J_{CW}=125 Hz), 142.6 (d, *J*=10 Hz), 142.2 (dd, *J*=9 Hz, *J*=5 Hz), 140.1 (s), 138.4 (d, *J*=3 Hz), 129.7 (s), 129.1 (s), 128.9 (s), 128.4 (s), 128.1 (s), 128.0 (s), 64.1 (d, *J*=6 Hz), 64.0 (d, *J*=6 Hz), 33.9 (d, *J*=21 Hz), 28.7 (d,

J=7 Hz), 15.7 (d, *J*=7 Hz), 15.6 (d, *J*=7 Hz) ppm. HRMS (solution in $CHCl_3$/ACN with addition of AgTFA): calc. for $C_{27}H_{30}O_9P_2WAg$, $[M+Ag]^+$ 852.99376, found 852.99413.

Preparation of compounds 8 and 9.

General procedure. Solution of phosphaallene **6a-d** prepared by method A, B or C was treated with excess of water or methanol. Reaction mixture stirred for 1h and extracted with diethyl ether (3×50ml), dried with brine and $MgSO_4$. Final products are obtained by column chromatography on silica gel using pentane/diethyl ether mixtures as eluent.

Compound 8a. Solution of phosphaallene prepared by method C from 795mg (1.40mmol) of **1**, 271mg (1.40mmol) of **3** and 1.4ml (1.40mmol) of 1M solution of *t*BuOLi in THF was treated with water and worked up as described above. Chromatography in 20% of diethyl ether in pentane gave final product (R_f=0.26). Yield: 501mg; 57%.

^{31}P NMR ($CDCl_3$): δ = 97.4 ($^1J_{PW}$=282 Hz) ppm. ^{1}H NMR ($CDCl_3$): δ = 7-55-7.25 (m, 13H, Ph), 7.09-7.07 (m, 2H, Ph), 6.91 (d, $^2J_{HP}$=24 Hz, 1H, H̲C=), 3.06 (bs, 1H, OH̲) ppm. ^{13}C NMR ($CDCl_3$): δ = 199.9 (d, *J*=24 Hz), 196.5 (d, J_{CP}=8 Hz, J_{CW}=126 Hz), 152.8 (d, *J*=4 Hz), 142.7 (d, *J*=44 Hz), 141.1 (d, *J*=11 Hz), 138.3 (d, *J*=6 Hz), 130.0 (d, *J*=2 Hz), 129.3 (s), 129.3 (s), 129.1 (s), 128.9 (s), 128.7 (d, *J*=10 Hz), 128.5 (s), 128.5 (d, *J*=11 Hz), 127.8 (s), 128.3 (d, *J*=13 Hz) ppm. HRMS (solution in $CHCl_3$ with addition of AgTFA): calc. for $C_{25}H_{17}O_6PWAg$, $[M+Ag]^+$ 736.93349, found 736.93393.

Compound 8b. Solution of phosphaallene prepared by method C from 581mg (1.09mmol) of **2**, 213mg (1.09mmol) of **3** and 1.09ml (1.09mmol) of 1M solution of tBuOLi in THF was treated with water and worked up as described above. Chromatography in 20% of diethyl ether in pentane gave final product (R_f=0.46). Yield: 350mg; 53%.

^{31}P NMR ($CDCl_3$): δ = 129.3 ($^1J_{PW}$=270 Hz) ppm. ^{1}H NMR ($CDCl_3$): δ = 7.46-7.42 (m, 2H, Ph), 7.35-7.30 (m, 6H, Ph), 7.25-7.22 (m, 2H, Ph), 6.45 (d, $^2J_{HP}$=34 Hz, 1H, H̲C=), 2.32 (bs, 1H, OH̲), 1.24 (d, $^3J_{HP}$=16 Hz, 9H, *t*Bu) ppm. ^{13}C NMR ($CDCl_3$): δ = 199.3 (d, *J*=24 Hz), 196.9 (d, J_{CP}=8 Hz, J_{CW}=126 Hz), 150.3 (d, *J*=5 Hz), 141.3 (d, *J*=10 Hz), 138.4 (d, *J*=5 Hz), 129.2 (d, *J*=1 Hz), 128.9 (s), 128.8 (s), 128.7 (s), 128.5 (s), 127.2 (s), 125.1 (d, *J*=24 Hz), 37.9 (d, *J*=27 Hz), 25.0 (d, *J*=6 Hz) ppm. HRMS (solution in $CHCl_3$/MeOH): calc. for $C_{23}H_{21}O_6PWNa$, $[M+Na]^+$ 631.04773, found 631.04824.

Compound 8c. Solution of phosphaallene prepared by method B from 100mg (0.176mmol) of **1**, 40mg (0.176mmol) of **S2** and 0.35ml (0.35mmol) of 1M solution of tBuOLi in THF was

treated with water and worked up as described above. Chromatography in pentane/diethyl ether 1:1 mixture gave final product (R_f=0.6). Yield: 34mg; 31%.

^{31}P NMR ($CDCl_3$): δ = 99.1 ($^{1}J_{PW}$=280 Hz) ppm. ^{1}H NMR ($CDCl_3$): δ = 7.88-7.80 (m, 2H, Ar), 7.77 (d, $^{3}J_{HH}$=8 Hz, 1H, Ar), 7.70 (d, $^{3}J_{HH}$=8 Hz, 1H, Ar), 7.67-7.60 (m, 2H, Ar), 7.56-7.46 (m, 3H, Ar), 7.42-7.29 (m, 3H, Ar), 7.16 (d, $^{2}J_{HP}$=16 Hz, 1H, $\underline{H}$C=), 7.16-7.10 (m, 1H, Ar), 4.54 (bs, 1H, O$\underline{H}$) ppm. ^{13}C NMR ($CDCl_3$): δ = 199.0 (d, J=24 Hz), 196.1 (d, J_{CP}=8 Hz, J_{CW}=126 Hz), 147.0 (d, J=2 Hz), 142.0 (s), 140.3 (s), 139.8 (d, J=44 Hz), 138.4 (d, J=13 Hz), 134.9 (d, J=3 Hz), 131.2 (d, J=2 Hz), 130.1 (d, J=2 Hz), 129.5 (d, J=14 Hz), 129.1 (s), 129.0 (s), 128.7 (s), 127.5 (s), 126.9 (s), 125.0 (d, J=35 Hz), 121.1 (s), 119.9 (s), 119.9 (s) ppm. HRMS (solution in $CHCl_3$ with addition of AgTFA): calc. for $C_{50}H_{30}O_{12}P_2W_2Ag$, $[2M+Ag]^+$ 1358.92743, found 1358.93151.

Compound 8d. Solution of phosphaallene prepared by method B from 50mg (0.094mmol) of **2**, 22mg (0.094mmol) of **S2** and 0.37ml (0.37mmol) of 1M solution of *t*BuOLi in THF was treated with water and worked up as described above. Chromatography in pentane/diethyl ether 1:1 mixture gave final product (R_f=0.69). Yield: 20mg; 35%.

^{31}P NMR (dcm-d_2): δ = 125.1 ($^{1}J_{PW}$=270 Hz) ppm. ^{1}H NMR (dcm-d_2): δ = 8.48 (d, $^{3}J_{HH}$=8 Hz, 1H, Ar), 7.75-7.63 (m, 3H, Ar), 7.46-7.36 (m, 2H, Ar), 7.36-7.27 (m, 2H, Ar), 6.89 (dd, $^{2}J_{HP}$=25 Hz, J_{HH}=2 Hz, 1H, $\underline{H}$C=), 1.35 (dd, $^{3}J_{HP}$=16 Hz, J_{HH}=2 Hz, 9H, *t*Bu) ppm. ^{13}C NMR (dcm-d_2): δ = 198.8 (d, J=24 Hz), 196.7 (d, J_{CP}=7 Hz, J_{CW}=126 Hz), 145.6 (d, J=4 Hz), 142.2 (s), 139.6 (s), 138.8 (d, J=11 Hz), 135.4 (d, J=3 Hz), 130.0 (s), 129.8 (s), 128.3 (s), 127.5 (s), 127.3 (s), 122.9 (d, J=27 Hz), 120.6 (s), 119.9 (s), 119.7 (s), 38.9 (d, J=28 Hz), 25.2 (d, J=6 Hz) ppm. HRMS (solution in $CHCl_3$/ACN with addition of AgTFA): calc. for $C_{23}H_{19}O_6PWAg$, $[M+Ag]^+$ 714.94912, found 714.94919.

Compound 9a. Solution of phosphaallene prepared by method C from 249mg (0.44mmol) of **1**, 85mg (0.44mmol) of **3** and 0.44ml (0.44mmol) of 1M solution of *t*BuOLi in THF was treated with methanol and worked up as described above. Chromatography in pentane gave final product (R_f=0.37). Yield: 153mmg; 54%.

^{31}P NMR (C_6D_6): δ = 115.0 ($^{1}J_{PW}$=280 Hz) ppm. ^{1}H NMR (C_6D_6): δ = 7.51-7.44 (m, 2H, Ph), 7.24-7.19 (m, 2H, Ph), 7.09-7.04 (m, 2H, Ph), 7.04-6.95 (m, 9H, Ph), 6.69 (d, $^{2}J_{HP}$=23 Hz, 1H, $\underline{H}$C=), 2.78 (d, $^{3}J_{HP}$=13 Hz, 3H, OC$\underline{H}_3$) ppm. ^{13}C NMR (C_6D_6): δ = 199.4 (d, J=24 Hz), 196.9 (d, J=8 Hz), 155.2 (d, J=2 Hz), 142.7 (d, J=12 Hz), 138.6 (d, J=43 Hz), 138.5 (d, J=4 Hz), 130.5 (d, J=2 Hz), 130.3 (s), 130.2 (s), 130.1 (s), 129.0 (s), 128.6 (s), 128.4 (d, J=5 Hz),

128.4 (s), 128.3 (s), 127.0 (d, J=38Hz), 53.7 (d, J=6 Hz) ppm. HRMS (solution in $CHCl_3$ /MeOH): calc. for $C_{26}H_{19}O_6PWNa$, $[M+Na]^+$ 665.03208, found 665.03235.

Compound 9b. Solution of phosphaallene prepared by method C from 0.144mg (0.27mmol) of **2**, 53mg (0.27mmol) of **3** and 0.27ml (0.27mmol) of 1M solution of *t*BuOLi in THF was treated with methanol and worked up as described above. Chromatography in pentane gave final product (R_f=0.8). Yield: 92mmg; 55%.

^{31}P NMR (C_6D_6): δ = 148.0 ($^1J_{PW}$=264 Hz) ppm. ^1H NMR (C_6D_6): δ = 7.33-7.29 (m, 2H, Ph), 7.21-7.18 (m, 2H, Ph), 7.08-6.97 (m, 6H, Ph), 6.37 (d, $^2J_{HP}$=35 Hz, 1H, $\underline{H}$C=), 2.47 (d, $^3J_{HP}$=13 Hz, 3H, OC$\underline{H}_3$), 1.03 (d, $^3J_{HP}$=15 Hz, 9H, *t*Bu) ppm. ^{13}C NMR (C_6D_6): δ = 198.4 (d. J=23 Hz), 197.3 (d, J_{CP}=7 Hz, J_{CW}=125 Hz), 151.0 (d, J=5 Hz), 142.9 (d, J=11 Hz), 139.4 (d, J=5 Hz), 130.0 (s), 128.6 (s), 128.5 (s), 127.7 (s), 127.6 (s) 127.5 (s), 126.6 (d, J=30 Hz), 38.0 (d, J=34 Hz), 25.2 (d, J=6 Hz) ppm. HRMS (solution in $CHCl_3$/MeOH): calc. for $C_{24}H_{23}O_6PWNa$, $[M+Na]^+$ 645.06338, found 645.06399.

X-ray data for compounds 5c, 7a, 8b and 8c

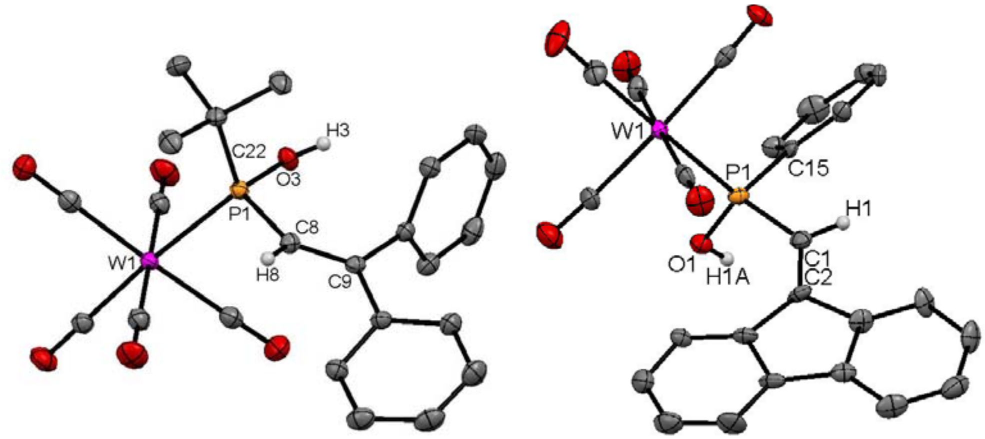

Figure ESI 1 Crystal structure of compounds **8b** (left) and **8c** (right) (ellipsoids set to 50% probability). All protons are omitted for clarity, except for that at the ^{III}P atom.

Table SI1. Crystallographic data for complex 5c, 7a, 8b and 8c.

Identification code		5c	7a	8b	8c
Empirical formula		$C_{29}H_{24}O_9P_2W_1$	$C_{29}H_{26}O_9P_2W_1$	$C_{23}H_{21}O_6P_1W_1$	$C_{25}H_{15}O_6P_1W_1$* C_4H_8O
Formula weight		762.27	764.29	608.22	698.29
Crystal description		yellow, block	colorless, plate	colorless block	yellow, block
Crystal size [mm]		0.22x0.30x0.50	0.08x0.11x0.13	0.25x0.30x0.42	0.25x0.25x0.30
Crystal system, space group		C 2/c	P 21/c	P -1	P 21/n
Unit cell dimensions:	a	20.4267(9)	12.0845(17)	9.466(9)	13.7467(10)
	b	9.4757(4)	11.3100(18)	10.519(9)	14.2640(9)
	c	30.355(1)	21.562(4)	12.887(12)	13.8844(9)
	α	90,	90	70.957(10)	90
	β	100.010(2)	93.790(9)	84.779(10)	97.278(5)
	γ	90	90	80.278(15)	90
Volume [Å^3]		5785.9(4)	2940.5(8)	1194.7(19)	2700.6(3)
Z		8	4	2	4
Calculated density [Mg/m^3]		1.750	1.726	1.691	1.717
F(000)		2992	1504	592	1368
Linear absorption coefficient μ		4.156	4.089	4.936	4.382
Absorption correction		Multi-scan			
Min. and max. transmission		0.2304/0.4616	0.5574/0.7455	0.2310/0.3717	0.0117/0.4071
Temperature		100(2)			
Diffractometer		Bruker D8 –APEX-II			
Radiation source		Mo K_α			
Radiation and wavelength		0.71073			
Scan type		ω-scan			
Range for data collection Θ		1.36-28.41	0.989-27.26	0.948-30.21	0.996-28.42
Index ranges		-23≥h≥27,<br>-12≥k≥9,<br>-40≥l≥40	-15≥h≥15,<br>-14≥k≥13,<br>-27≥l≥27	-13≥h≥13,<br>-14≥k≥14,<br>-18≥l≥16	-18≥h≥15,<br>-19≥k≥19,<br>-18≥l≥18
Reflections collected / unique		27328, 7265	24080, 6536	21852, 6735	26684, 6777
Significant unique reflections		5647	4890	6453	4584
R(int), R(sigma)		0.0572, 0.0595	0.0953, 0.0891	0.0339, 0.0307	0.1131, 0.1176
Completeness		0.998	0.989	0.948	0.996
Θ-max		28.41	27.26	30.21	28.42
Refinement method		Full-matrix least-squares on F^2			
Data / parameters / restraints		7265/359/1	6536/382/0	6735/293/0	6777/347/13
Goodness-of-fit on F^2		1.137	1.008	1.065	1.031
Final R indices [I > (I)]σ2		0.0486	0.0452	0.0187	0.0589
R indices (all data)		0.2148	0.1156	0.0455	0.2320
Weighting scheme		$w=1/[\sigma^2(F_o^2)+(aP)^2+bP]$ where $P=(F_o^2+2F_c^2)/3$			
Weighting scheme parameters a, b		0.1412, 1.1019	0.0462, 0.0000	0.0161, 0.5387	0.1442, 0.0000
Largest σ/Δ in last cycle		0.015	0.001	0.003	0.001
Largest difference peak and hole		1.953/-4.234	1.356/ -2.004	1.913/-1.011	1.905/-3.562
Structure Solution Program		ShelXS-97			
Structure Refinement Program		ShelXL-97			

Summary of selected bond lengths and angles of compound 5c.

W1-P1	2.468(2)
W1-C25	2.069(10)
W1-C26	2.041(9)
W1-C27	2.069(9)
W1-C28	2.057(9)
W1-C29	2.061(9)
P1-O1	1.649(6)
P1-C1	1.794(8)
P1-C19	1.820(9)
P2-O1	1.601(6)
P2-O2	1.560(7)
P2-O3	1.566(7)
P2-O4	1.460(6)
O2-C15	1.610(14)
O3-C17	1.556(15)
O5-C25	1.126(13)
O6-C26	1.118(11)
O7-C27	1.112(11)
O8-C28	1.115(11)
O9-C29	1.123(11)
C1-C2	1.343(12)
C2-C3	1.463(11)
C2-C14	1.466(11)
C3-C4	1.397(11)
C3-C8	1.407(12)
C4-C5	1.406(13)
C5-C6	1.373(13)
C6-C7	1.371(12)

P1 -W1-C25	90.4(3)
P1 -W1-C26	174.5(3)
P1 -W1-C27	92.0(3)
P1 -W1-C28	88.6(3)
P1 -W1-C29	85.8(3)
C25-W1-C26	93.8(4)
C25-W1-C27	88.8(4)
C25-W1-C28	178.9(4)
C25-W1-C29	88.1(4)
C26-W1-C27	91.7(4)
C26-W1-C28	87.3(4)
C26-W1-C29	90.7(4)
C27-W1-C28	91.1(4)
C27-W1-C29	176.2(4)
C28-W1-C29	92.0(3)
W1 -P1-O1	106.2(2)
W1 -P1-C1	116.8(3)
W1 -P1-C19	121.5(3)
O1 -P1-C1	100.3(3)
O1 -P1-C19	102.2(3)

C25-W1-P1 -O1	56.0(3)
C25-W1-P1 -C1	166.9(4)
C25-W1-P1 -C19	-59.9(4)
C27-W1-P1 -O1	144.8(4)
C27-W1-P1 -C1	-104.3(4)
C27-W1-P1 -C19	28.9(4)
C28-W1-P1 -O1	-124.2(3)
C28-W1-P1 -C1	-13.3(4)
C28-W1-P1 -C19	120.0(4)
C29-W1-P1 -O1	-32.1(3)
C29-W1-P1 -C1	78.8(4)
C29-W1-P1 -C19	-148.0(4)
W1 -P1-O1 -P2	176.8(4)
C1 -P1-O1 -P2	54.7(6)
C19-P1-O1 -P2	-55.0(6)
W1 -P1-C1 -C2	71.6(9)
O1 -P1-C1 -C2	-174.1(8)
C19-P1-C1 -C2	-68.0(9)
W1 -P1-C19-C20	29.5(8)
W1 -P1-C19-C24	-154.2(6)
O1 -P1-C19-C20	-88.4(7)
O1 -P1-C19-C24	88.0(7)
C1 -P1-C19-C20	166.8(7)
C1 -P1-C19-C24	-16.9(8)
O2 -P2-O1 -P1	91.3(5)
O3 -P2-O1 -P1	-159.9(5)
O4 -P2-O1 -P1	-33.4(6)
O1 -P2-O2 -C15	37.1(8)

Summary of selected bond lengths and angles of compound 7a.

W1-P1	2.5066(16)
W1-C25	2.005(6)
W1-C26	2.036(6)
W1-C27	2.034(7)
W1-C28	2.065(7)
W1-C29	2.057(7)
P1-C1	1.828(5)
P1-C19	1.828(6)
P2-O1	1.586(5)
P2-O2	1.553(5)
P2-O3	1.553(5)
P2-O4	1.452(5)
P1-H1	1.28(7)
O1-C1	1.417(7)
O2-C17A	1.493(11)
O2-C17B	1.51(4)
O3-C15	1.470(9)
O5-C25	1.155(8)
O6-C26	1.154(8)
O7-C27	1.147(8)
O8-C28	1.128(8)
O9-C29	1.124(8)
C1-C2	1.325(8)
C2-C3	1.492(8)
C2-C14	1.488(9)
C3-C8	1.410(9)
C3-C4	1.383(9)
C4-C5	1.409(9)

P1 -W1-C25	172.82(17)
P1 -W1-C26	91.97(18)
P1 -W1-C27	91.13(19)
P1 -W1-C28	93.12(18)
P1 -W1-C29	84.56(18)
C25-W1-C26	89.3(2)
C25-W1-C27	87.7(3)
C25-W1-C28	94.0(2)
C25-W1-C29	88.3(2)
C26-W1-C27	176.9(3)
C26-W1-C28	88.2(3)
C26-W1-C29	91.8(3)
C27-W1-C28	91.0(3)
C27-W1-C29	89.1(3)
C28-W1-C29	177.7(2)
W1 -P1-C1	113.2(2)
W1 -P1-C19	125.7(2)
C1 -P1-C19	106.7(3)
O1 -P2-O2	102.2(2)
O1 -P2-O3	101.3(2)

C26-W1-P1 -C1	178.2(3)
C27-W1-P1 -C1	-2.4(3)
C28-W1-P1 -C1	-93.5(3)
C29-W1-P1 -C1	86.6(3)
C26-W1-P1 -C19	-47.9(3)
C27-W1-P1 -C19	131.5(3)
C28-W1-P1 -C19	40.5(3)
C29-W1-P1 -C19	-139.5(3)
C1 -P1-C19-C20	168.3(5)
W1 -P1-C19-C24	-152.2(4)
C1 -P1-C19-C24	-15.9(6)
C19-P1-C1 -O1	-88.5(5)
W1 -P1-C1 -C2	-111.4(6)
C19-P1-C1 -C2	106.3(6)
W1 -P1-C1 -O1	53.8(4)
W1 -P1-C19-C20	32.1(6)
O4 -P2-O3 -C15	42.6(6)
O1 -P2-O3 -C15	-82.9(5)
O1 -P2-O2 -C17A	175.6(6)
O3 -P2-O2 -C17A	-79.8(6)
O4 -P2-O2 -C17A	48.7(7)
O3 -P2-O1 -C1	169.5(4)
O2 -P2-O3 -C15	171.8(5)
O2 -P2-O1 -C1	-85.3(4)

Summary of selected bond lengths and angles of compound 8b.

W1 -P1	2.542(2)	P1-W1-C1	83.44(6)	C1 -W1-P1 -O3	61.86(9)
W1 -C1	2.079(3)	P1-W1-C3	91.54(6)	C1 -W1-P1 -C8	-55.85(9)
W1 -C3	2.064(3)	P1-W1-C4	89.30(6)	C1 -W1-P1 -C22	-175.90(10)
W1 -C4	2.086(3)	P1-W1-C6	95.48(6)	C3 -W1-P1 -O3	153.39(9)
W1 -C6	2.072(3)	P1-W1-C26	175.91(6)	C3 -W1-P1 -C8	35.69(9)
W1 -C26	2.037(3)	C1-W1-C3	91.70(9)	C3 -W1-P1 -C22	-84.36(10)
P1 -O3	1.648(2)	C1-W1-C4	87.89(9)	C4 -W1-P1 -O3	-26.10(9)
P1 -C8	1.821(3)	C1-W1-C6	177.39(8)	C4 -W1-P1 -C8	-143.81(9)
P1 -C22	1.883(3)	C1-W1-C26	92.53(8)	C4 -W1-P1 -C22	96.15(10)
O1 -C3	1.159(3)	C3-W1-C4	179.02(8)	C6 -W1-P1 -O3	-115.76(9)
O2 -C6	1.164(3)	C3-W1-C6	90.70(8)	C6 -W1-P1 -C8	126.54(9)
O4 -C1	1.160(3)	C3-W1-C26	87.88(8)	C6 -W1-P1 -C22	6.49(10)
O5 -C4	1.155(3)	C4-W1-C6	89.73(8)	W1 -P1-C8 -C9	109.97(19)
O6 -C26	1.165(3)	C4-W1-C26	91.25(8)	O3 -P1-C8 -C9	-7.7(2)
O3 -H3	0.8400	C6-W1-C26	88.58(8)	C22-P1-C8 -C9	-119.9(2)
C8 -C9	1.374(3)	W1-P1-O3	108.29(6)	C8 -P1-C22-C24	55.92(16)
C9 -C10	1.523(3)	W1-P1-C8	109.00(7)	W1 -P1-C22-C25	-62.68(16)
C9 -C16	1.493(3)	W1-P1-C22	121.00(7)	O3 -P1-C22-C25	60.74(16)
C10-C15	1.408(3)	O3-P1-C8	108.38(9)	C8 -P1-C22-C25	174.76(15)
C10-C11	1.425(3)	O3-P1-C22	105.82(9)	W1 -P1-C22-C23	57.27(16)
C11-C12	1.415(4)	C8-P1-C22	103.78(9)	O3 -P1-C22-C23	-179.31(14)
C12-C13	1.391(4)	P1-O3-H3	109.00	C8 -P1-C22-C23	-65.30(16)
C13-C14	1.416(4)	W1-C1-O4	179.10(18)	W1 -P1-C22-C24	178.49(11)
C14-C15	1.426(3)	W1-C3-O1	176.78(18)	O3 -P1-C22-C24	-58.09(16)
C16-C17	1.424(3)	W1-C4-O5	179.04(18)	P1 -C8-C9 -C10	16.3(3)
C16-C21	1.436(3)	W1-C6-O2	178.21(18)	P1 -C8-C9 -C16	-166.61(15)
C17-C18	1.410(4)	P1-C8-C9	130.71(16)	C16-C9-C10-C11	74.2(2)
		C8-C9-C10	123.01(18)	C16-C9-C10-C15	-105.6(2)

Summary of selected bond lengths and angles of compound 8c.

W1 -P1	2.493(3)	P1 -W1 -C21	92.2(3)		
W1 -C21	2.060(12)	P1 -W1 -C22	84.5(3)	C21-W1 -P1 -O6	156.8(5)
W1 -C22	2.062(12)	P1 -W1 -C23	176.8(4)	C22-W1 -P1 -O6	67.8(5)
W1 -C23	1.996(11)	P1 -W1 -C24	89.6(3)	C24-W1 -P1 -O6	-20.8(5)
W1 -C24	2.056(12)	P1 -W1 -C25	91.4(3)	C25-W1 -P1 -O6	-111.1(5)
W1 -C25	2.085(12)	C21-W1 -C22	89.3(5)	C21-W1 -P1 -C1	-75.7(5)
P1 -O6	1.565(7)	C21-W1 -C23	87.6(5)	C22-W1 -P1 -C1	-164.7(6)
P1 -C1	1.773(11)	C21-W1 -C24	177.0(5)	C24-W1 -P1 -C1	106.7(6)
P1 -C15	1.824(10)	C21-W1 -C25	92.0(4)	C25-W1 -P1 -C1	16.4(5)
O1 -C21	1.114(15)	C22-W1 -C23	92.3(5)	C21-W1 -P1 -C15	38.1(5)
O2 -C22	1.116(15)	C22-W1 -C24	88.5(5)	C22-W1 -P1 -C15	-51.0(5)
O3 -C23	1.169(14)	C22-W1 -C25	175.8(4)	C24-W1 -P1 -C15	-139.5(5)
O4 -C24	1.124(15)	C23-W1 -C24	90.5(5)	C25-W1 -P1 -C15	130.1(5)
O5 -C25	1.119(15)	C23-W1 -C25	91.8(5)	W1 -P1 -C1 -C2	-69.1(12)
O6 -H6A	0.8400	C24-W1 -C25	90.4(5)	O6 -P1 -C1 -C2	59.4(11)
O7A-C33A	1.55(4)	W1 -P1 -O6	110.1(3)	C15-P1 -C1 -C2	168.3(11)
O7A-C30	1.46(3)	W1 -P1 -C1	121.5(4)	C1 -P1 -C15-C16	53.6(10)
O7B-C30	1.47(2)	W1 -P1 -C15	113.8(3)	W1 -P1 -C15-C20	100.5(8)
O7B-C33B	1.54(4)	O6 -P1 -C1	107.9(5)	O6 -P1 -C15-C20	-20.6(10)
C1 -C2	1.379(15)	O6 -P1 -C15	106.0(4)	C1 -P1 -C15-C20	-131.2(9)
C2 -C14	1.487(15)	C1 -P1 -C15	96.0(5)	W1 -P1 -C15-C16	-74.7(10)
C2 -C3	1.511(16)	P1 -O6 -H6A	109.00		
C3 -C4	1.368(16)	C30-O7A-C33A	116(3)		
C3 -C8	1.411(16)	C30-O7B-C33B	114(3)		
C4 -C5	1.392(18)	P1 -C1 -C2	131.3(8)		
C5 -C6	1.355(19)	C1 -C2 -C14	132.3(10)		
C6 -C7	1.376(19)	C1 -C2 -C3	121.0(10)		
C7 -C8	1.398(18)	C3 -C2 -C14	106.7(9)		

NMR data for complex 5a (^{1}H, ^{31}P, ^{13}C)

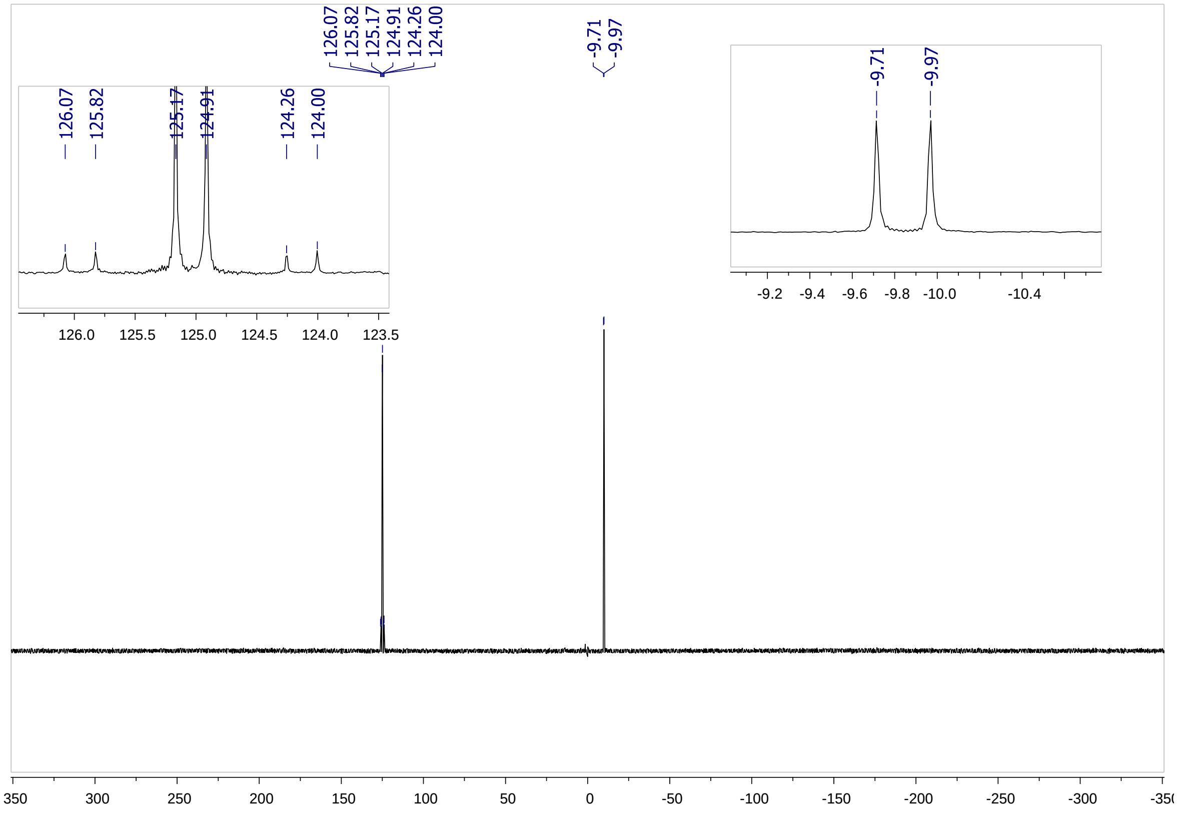

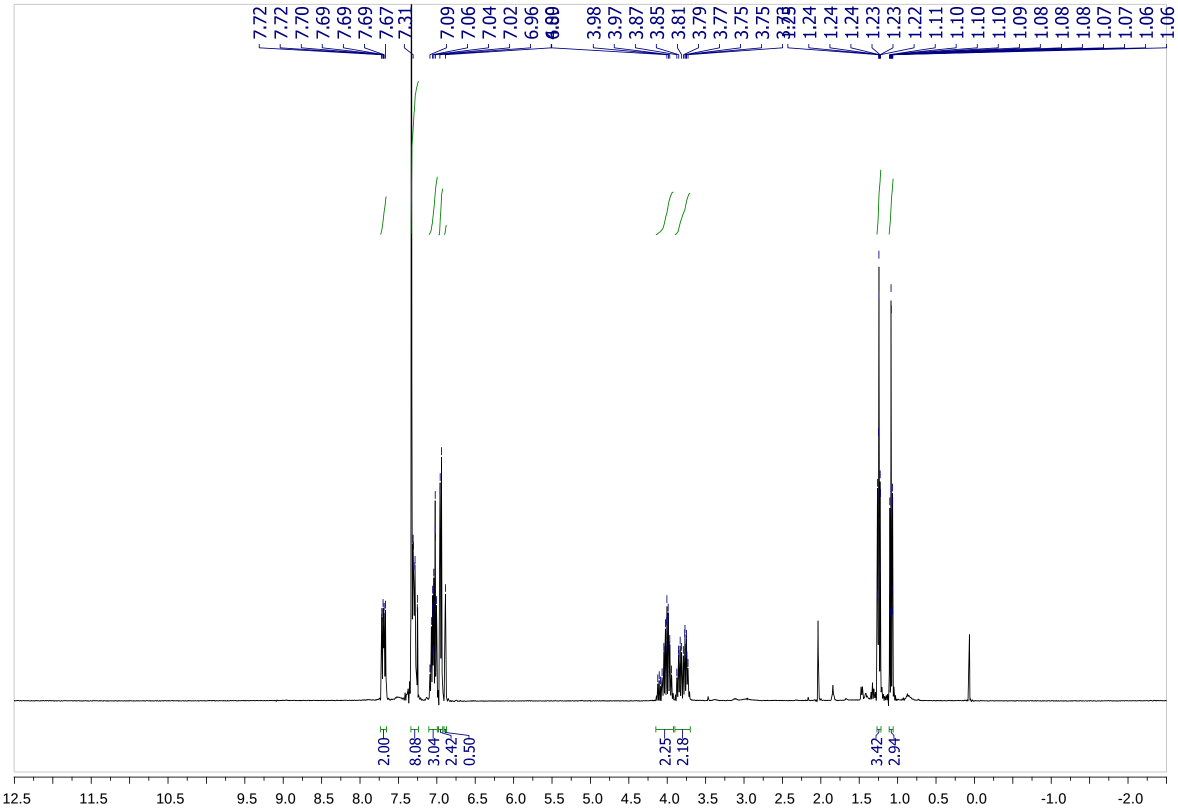
7.72
7.72
7.70
7.69
7.69
7.69
7.67
7.31
7.09
7.06
7.04
7.02
6.96
3.98
3.97
3.87
3.85
3.81
3.79
3.77
3.75
3.75
1.24
1.24
1.24
1.23
1.23
1.22
1.11
1.10
1.10
1.09
1.08
1.08
1.08
1.07
1.06
1.06
2.00
8.08
3.04
2.42
0.50
2.25
2.18
3.42
2.94
12.5
11.5
10.5
9.5
9.0
8.5
8.0
7.5
7.0
6.5
6.0
5.5
5.0
4.5
4.0
3.5
3.0
2.5
2.0
1.5
1.0
0.5
0.0
-1.0
-2.0

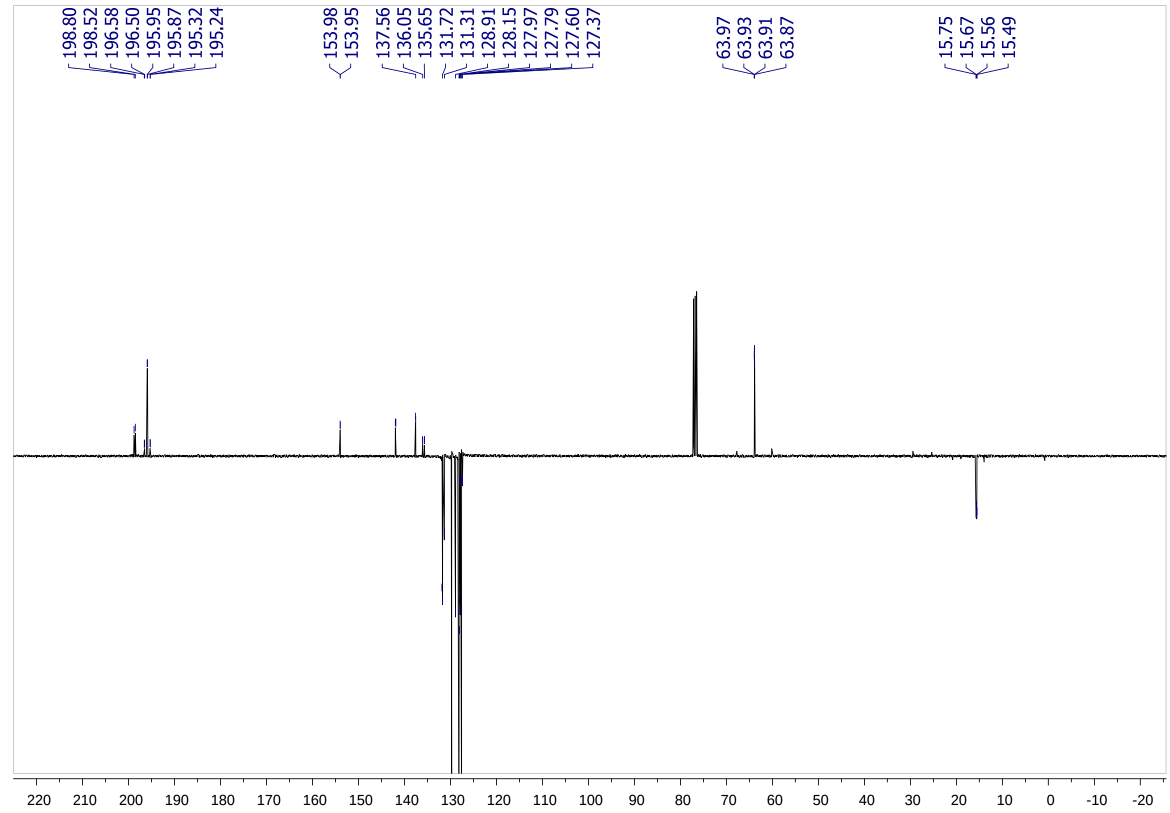
198.80
198.52
196.58
196.50
195.95
195.87
195.32
195.24
153.98
153.95
137.56
136.05
135.65
131.72
131.31
128.91
128.15
127.97
127.79
127.60
127.37
63.97
63.93
63.91
63.87
15.75
15.67
15.56
15.49
220
210
200
190
180
170
160
150
140
130
120
110
100
90
80
70
60
50
40
30
20
10
0
-10
-20

NMR data for complex 5b (^{1}H, ^{31}P, ^{13}C)

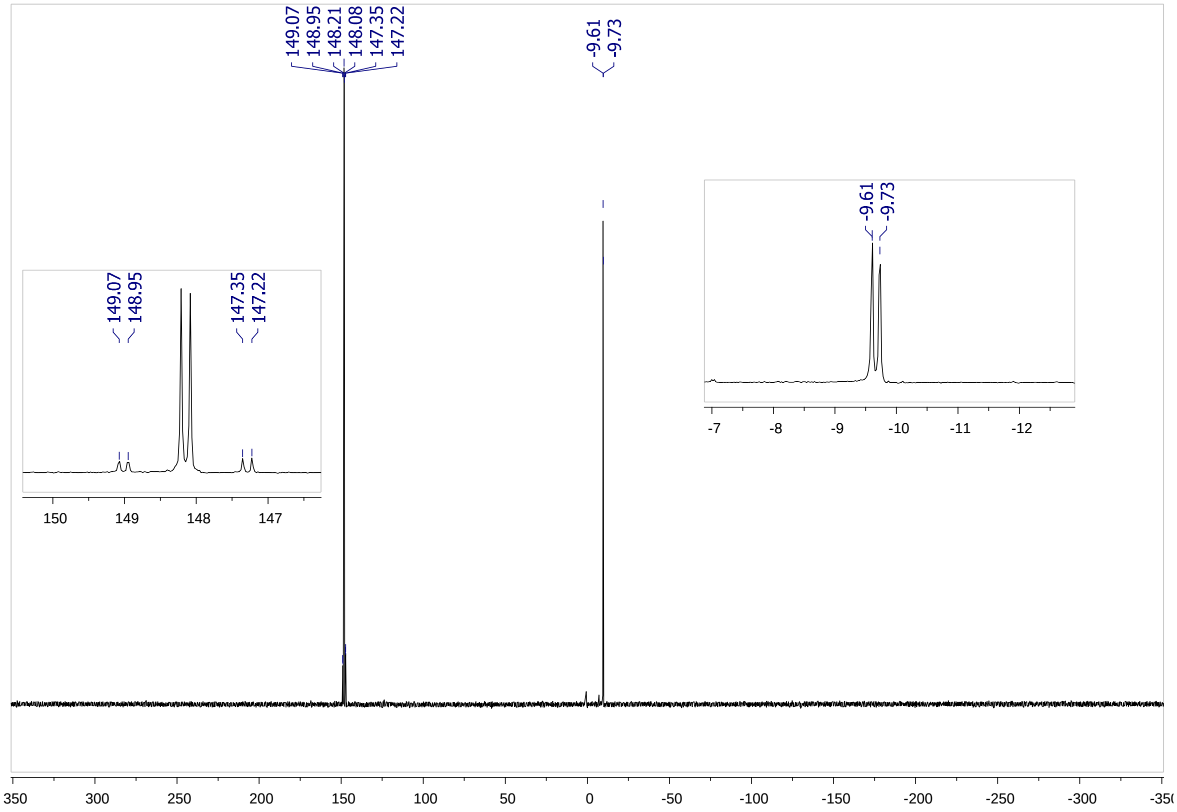

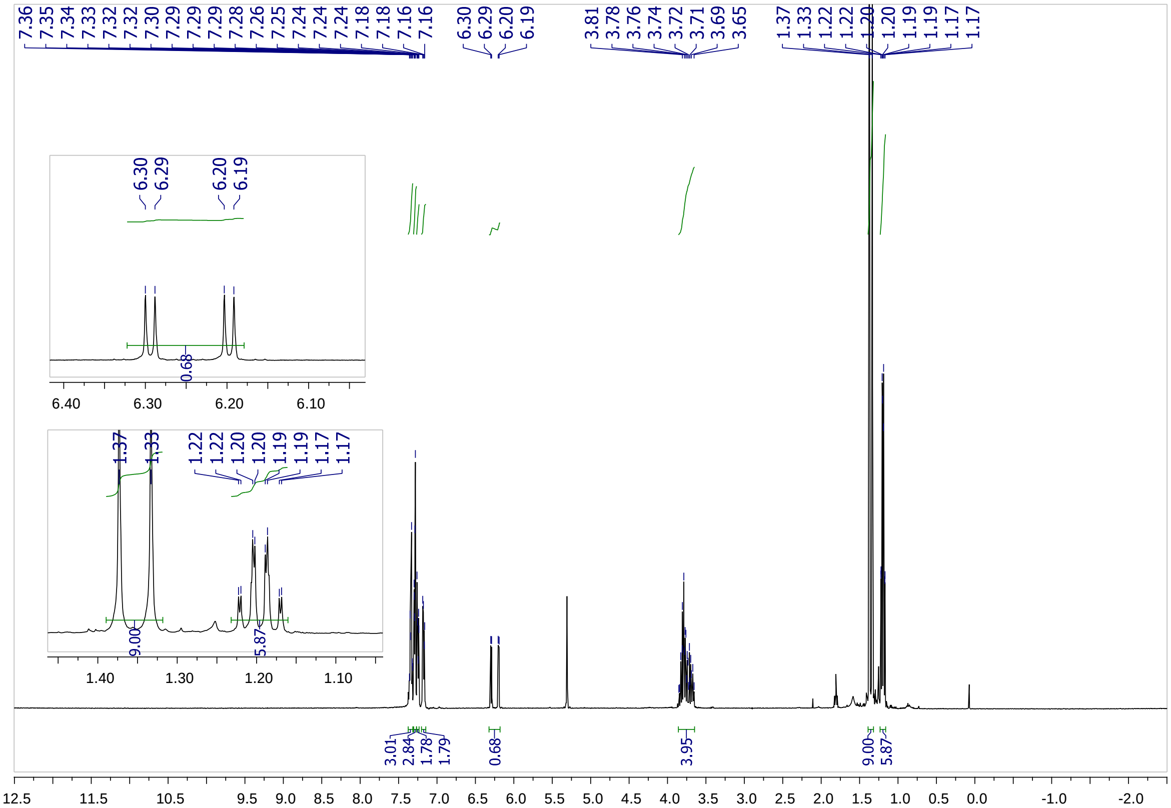

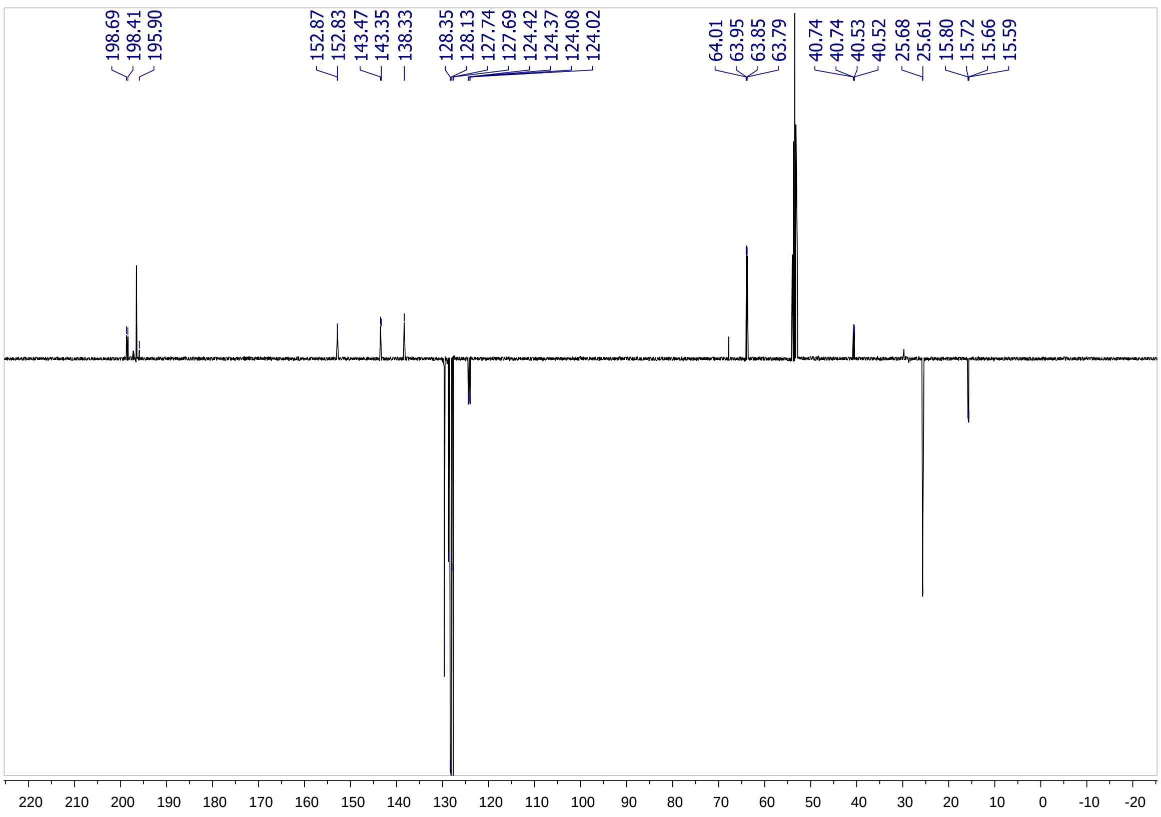

NMR data for complex 5c (^{1}H, ^{31}P, ^{13}C)

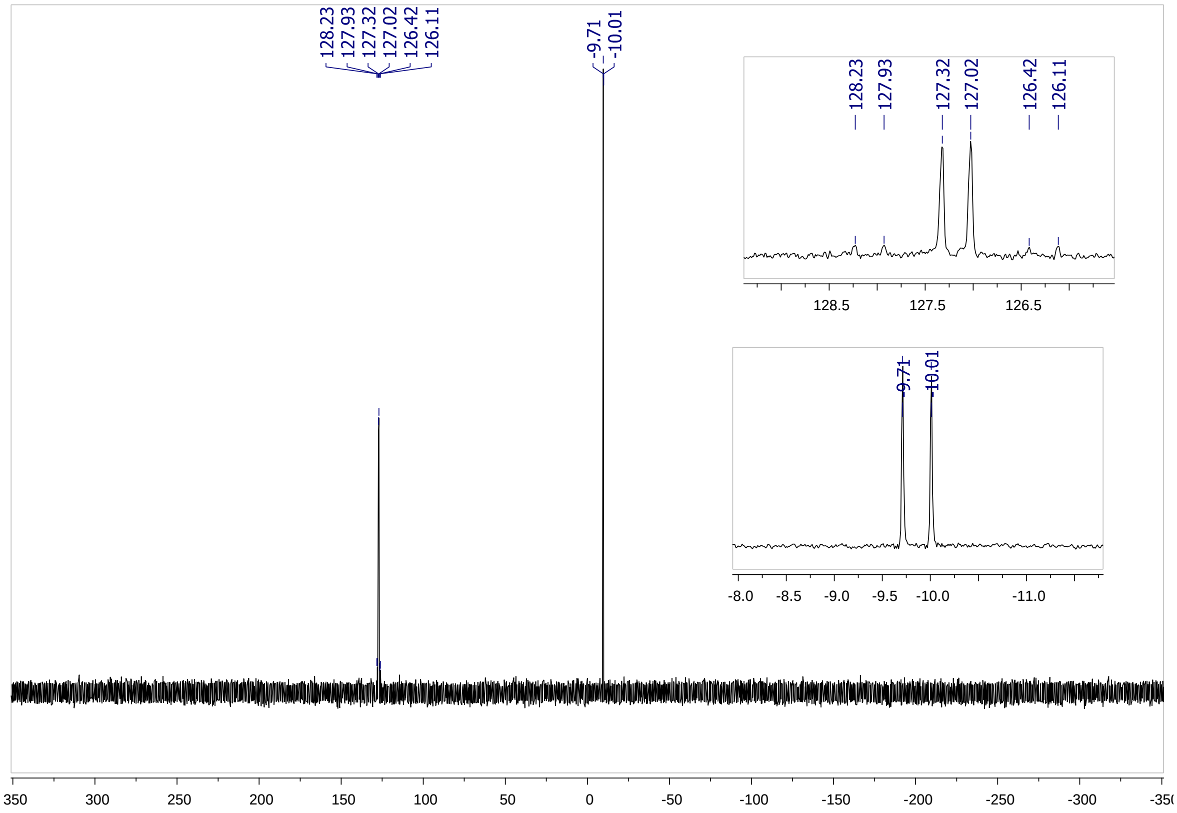

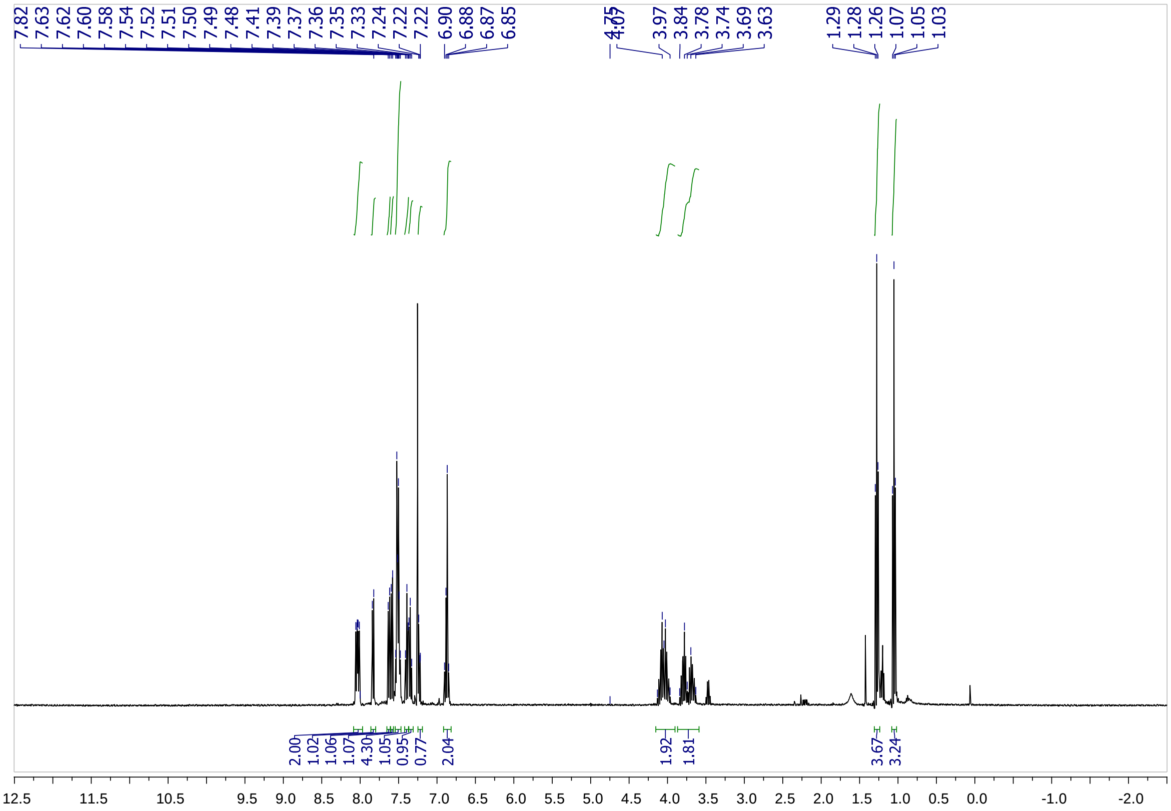
7.82
7.63
7.62
7.60
7.58
7.54
7.52
7.51
7.50
7.49
7.48
7.41
7.39
7.37
7.36
7.35
7.33
7.24
7.22
7.22
6.90
6.88
6.87
6.85
4.75
4.07
3.97
3.84
3.78
3.74
3.69
3.63
1.29
1.28
1.26
1.07
1.05
1.03
2.00
1.02
1.06
1.07
4.30
1.05
0.95
0.77
2.04
1.92
1.81
3.67
3.24
12.5 11.5 10.5 9.5 9.0 8.5 8.0 7.5 7.0 6.5 6.0 5.5 5.0 4.5 4.0 3.5 3.0 2.5 2.0 1.5 1.0 0.5 0.0 -1.0 -2.0

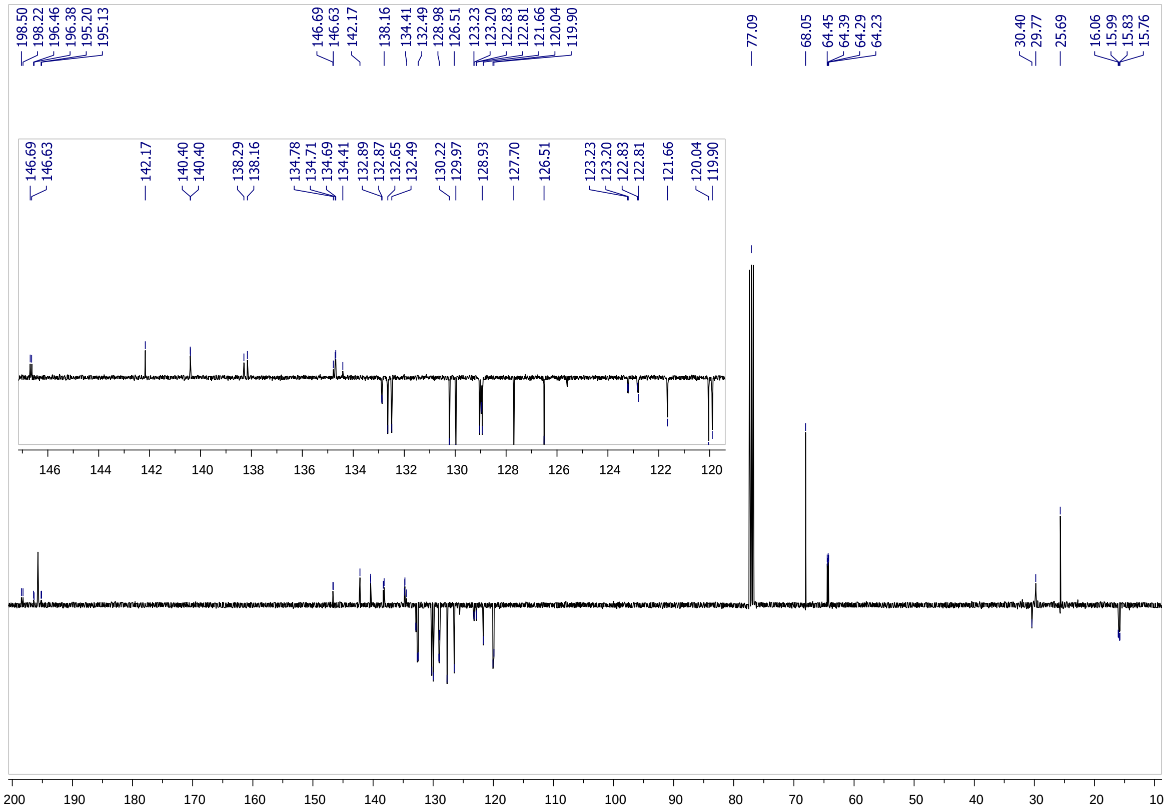
198.50
198.22
196.46
196.38
195.20
195.13
146.69
146.63
142.17
138.16
134.41
132.49
128.98
126.51
123.23
123.20
122.83
122.81
121.66
120.04
119.90
77.09
68.05
64.45
64.39
64.29
64.23
30.40
29.77
25.69
16.06
15.99
15.83
15.76
146.69
146.63
142.17
140.40
140.40
138.29
138.16
134.78
134.71
134.69
134.41
132.89
132.87
132.65
132.49
130.22
129.97
128.93
127.70
126.51
123.23
123.20
122.83
122.81
121.66
120.04
119.90
146 144 142 140 138 136 134 132 130 128 126 124 122 120
200 190 180 170 160 150 140 130 120 110 100 90 80 70 60 50 40 30 20 10

NMR data for complex 5d (^{1}H, ^{31}P, ^{13}C)

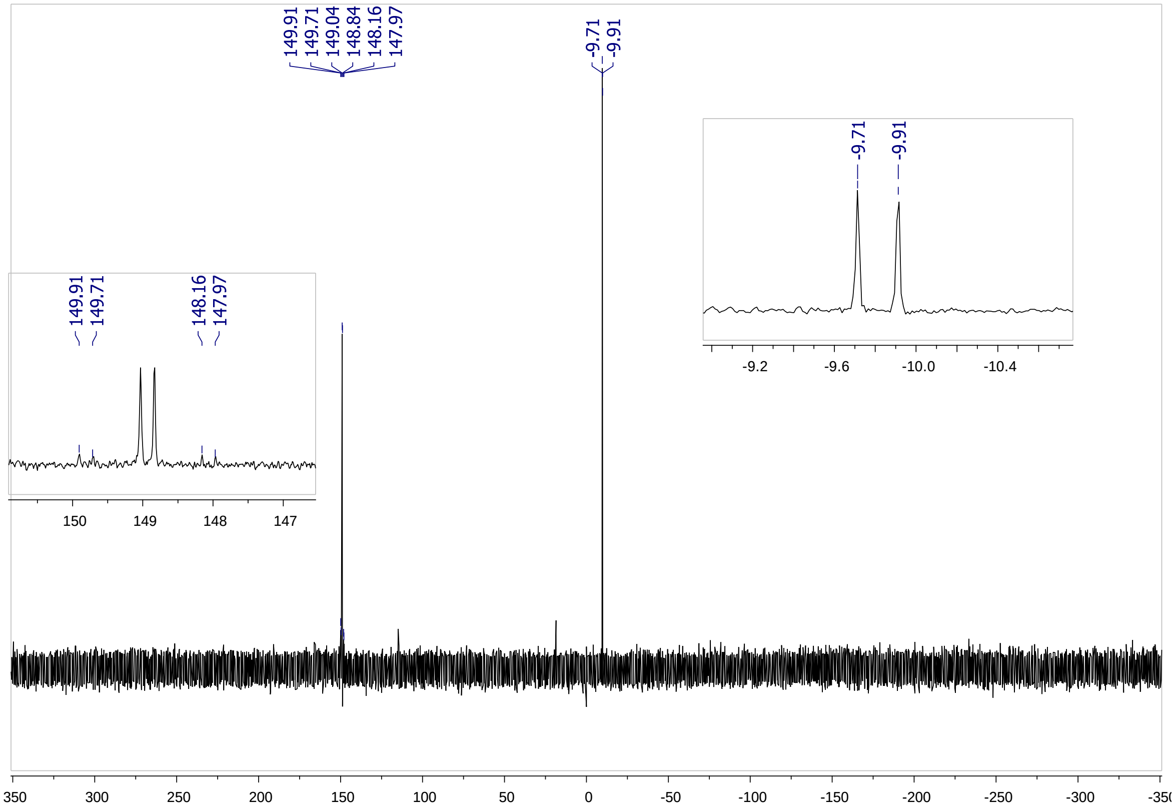

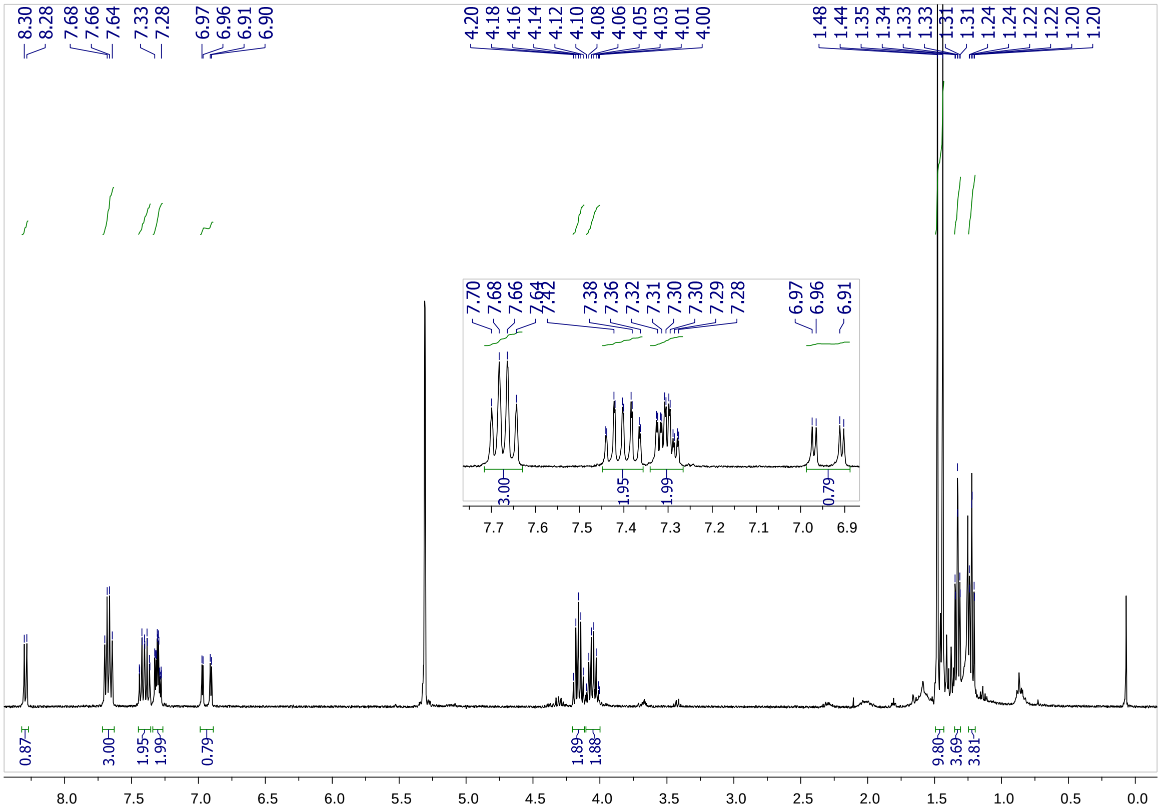

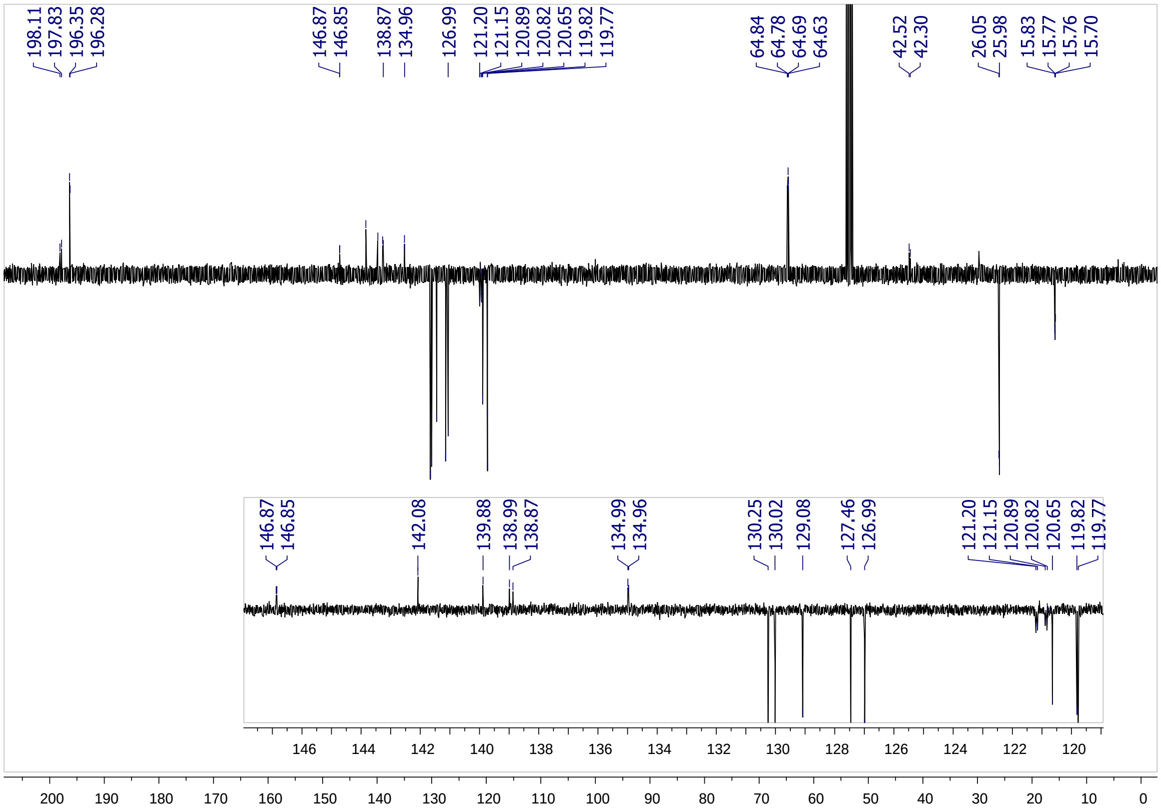

NMR data for complex 6a (^{31}P)

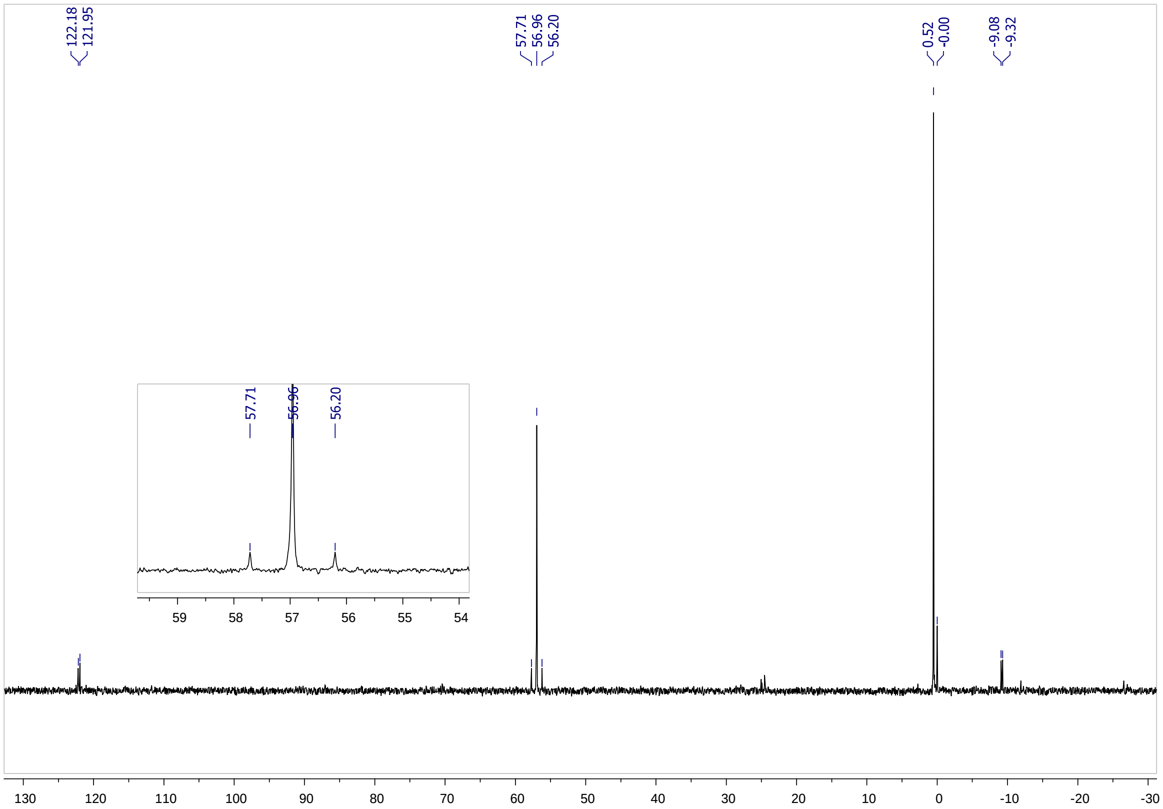

NMR data for complex 6b (^{31}P)

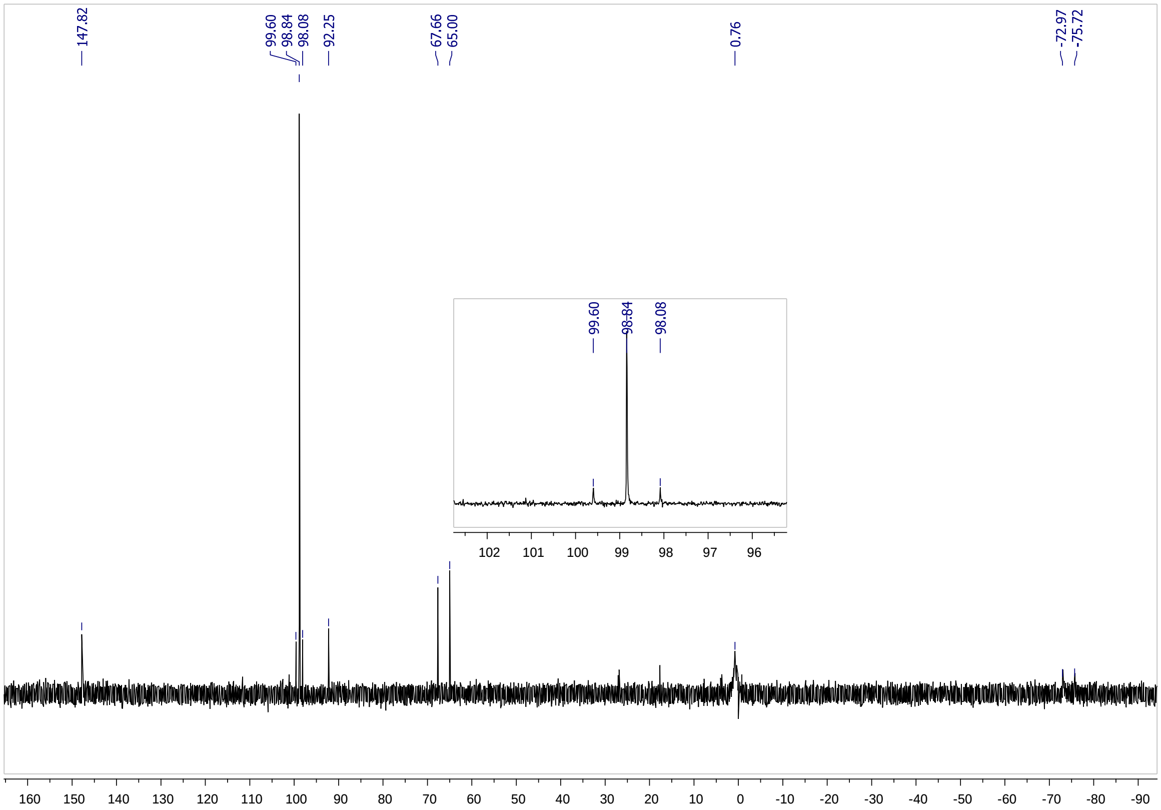

NMR data for complex 6c (^{31}P)

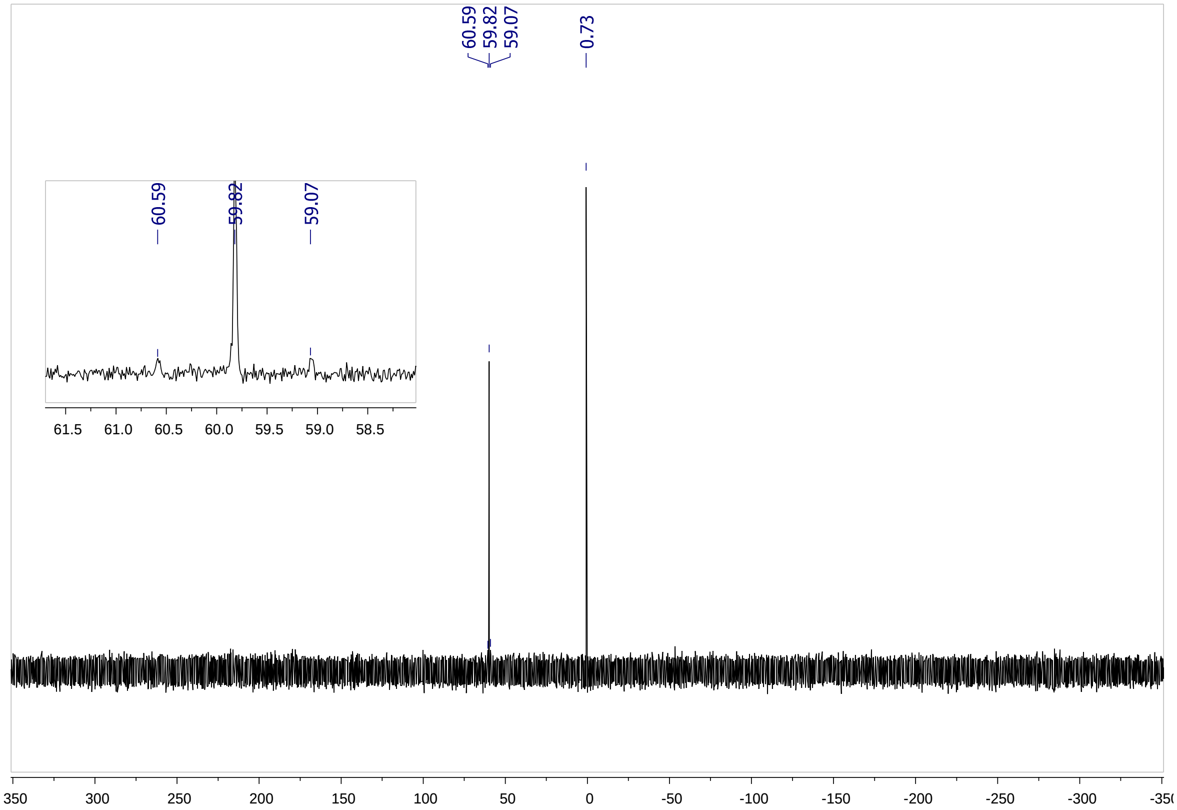

NMR data for complex 6d (^{31}P)

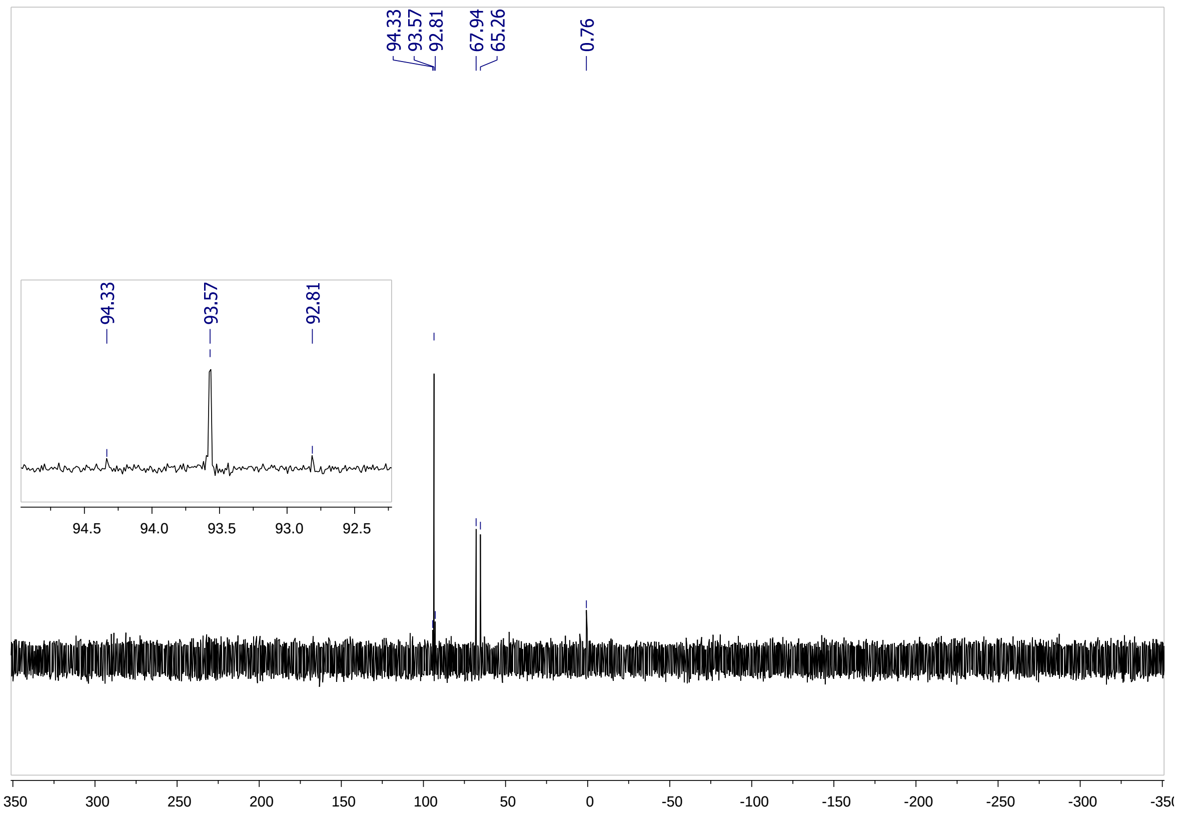

NMR data for complex 7a (^{1}H, ^{31}P, ^{13}C)

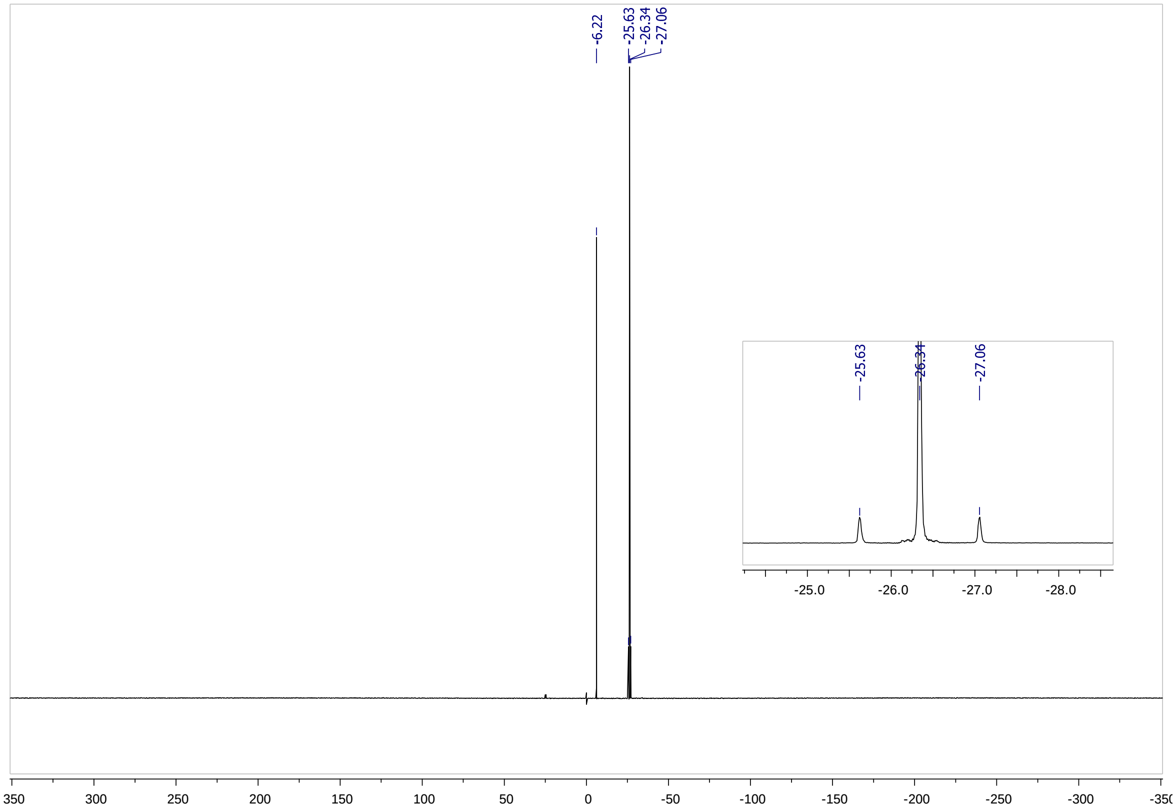

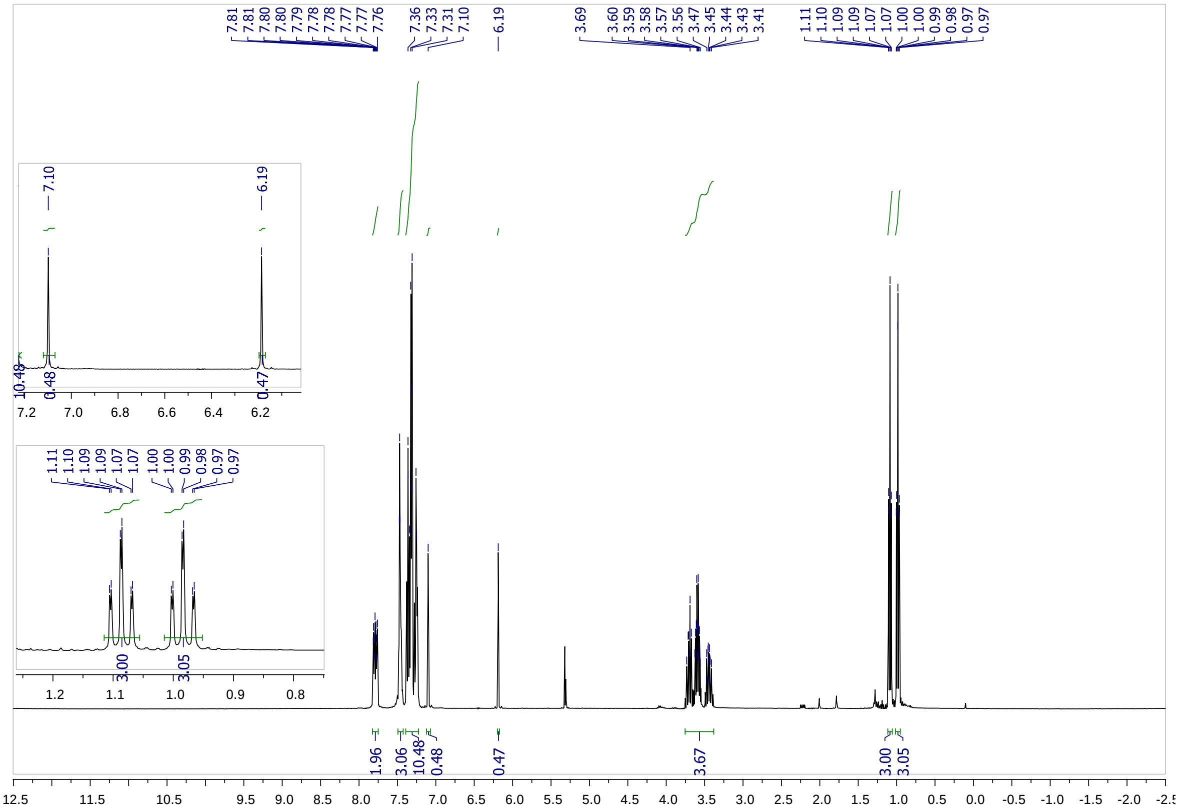

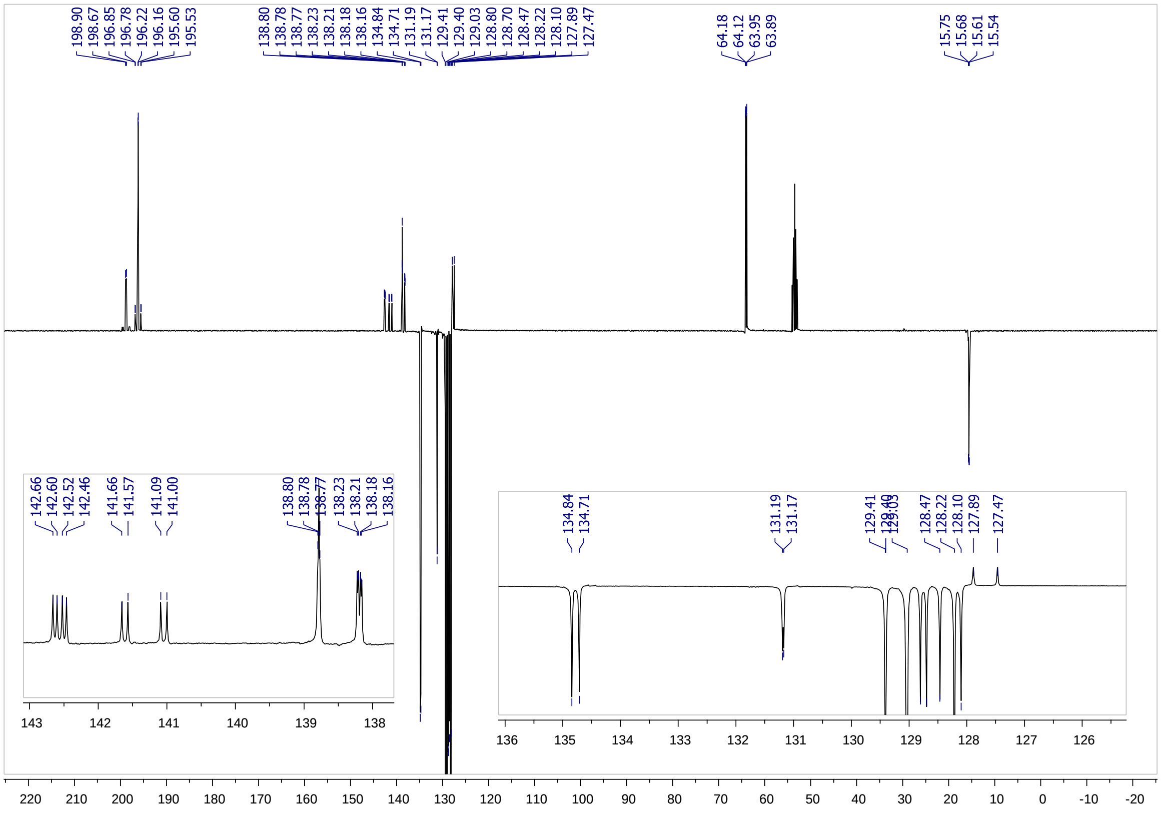

NMR data for complex 7b (^{1}H, ^{31}P, ^{13}C)

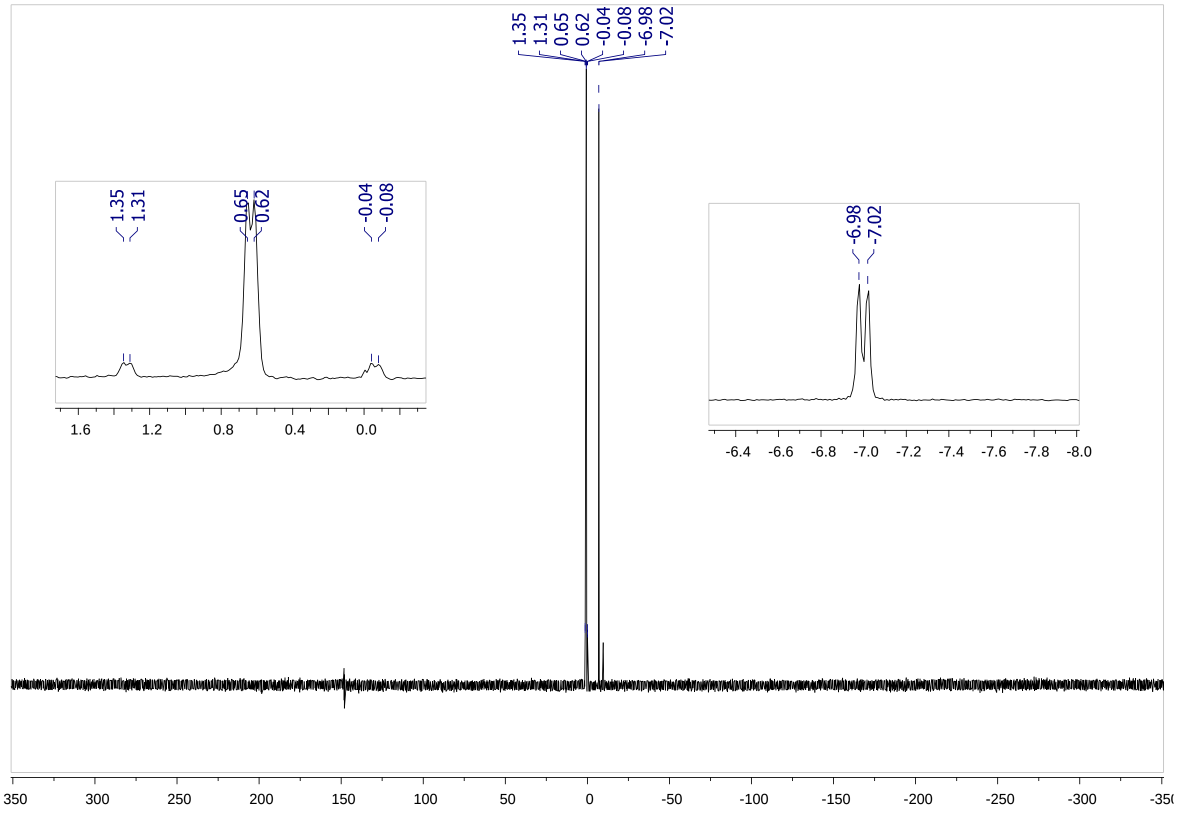

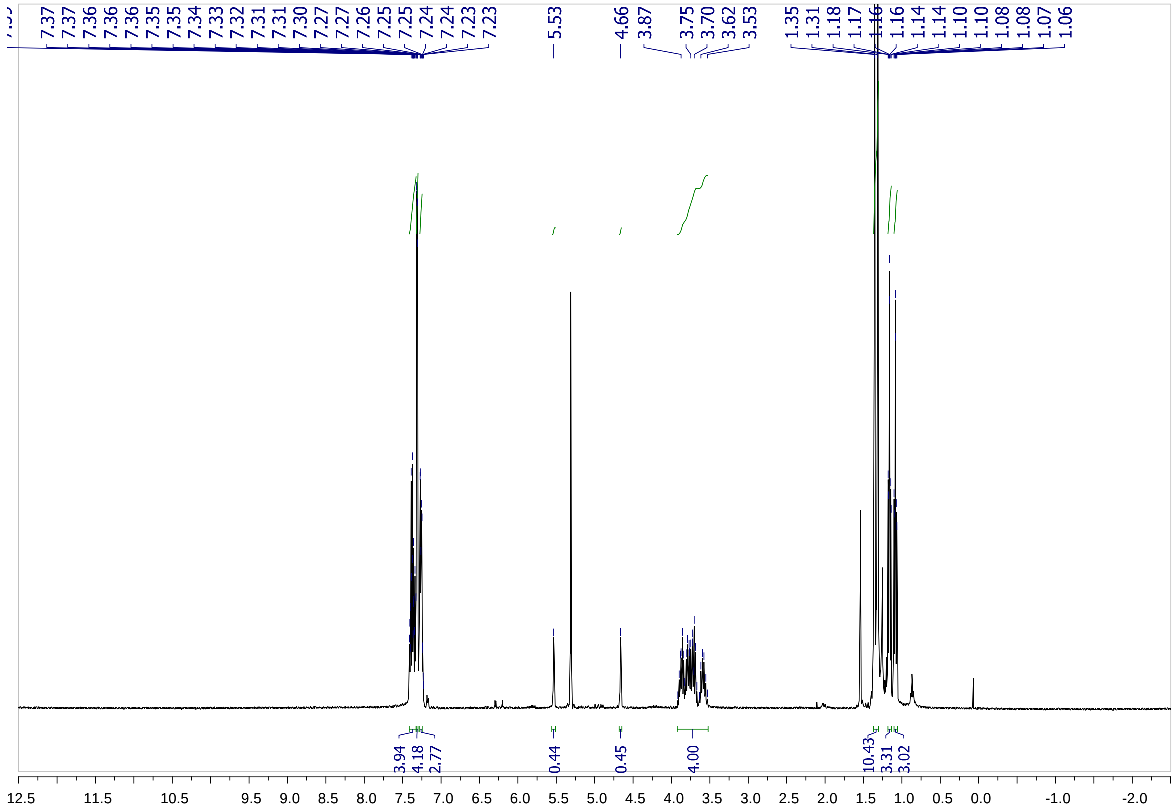
7.37 7.37 7.36 7.36 7.36 7.35 7.35 7.34 7.33 7.32 7.31 7.31 7.30 7.27 7.27 7.26 7.25 7.25 7.24 7.24 7.23 7.23
5.53
4.66
3.87 3.75 3.70 3.62 3.53
1.35 1.31 1.18 1.17 1.16 1.16 1.14 1.14 1.10 1.10 1.08 1.08 1.07 1.06
3.94 4.18 2.77
0.44
0.45
4.00
10.43 3.31 3.02
12.5 11.5 10.5 9.5 9.0 8.5 8.0 7.5 7.0 6.5 6.0 5.5 5.0 4.5 4.0 3.5 3.0 2.5 2.0 1.5 1.0 0.5 0.0 -1.0 -2.0

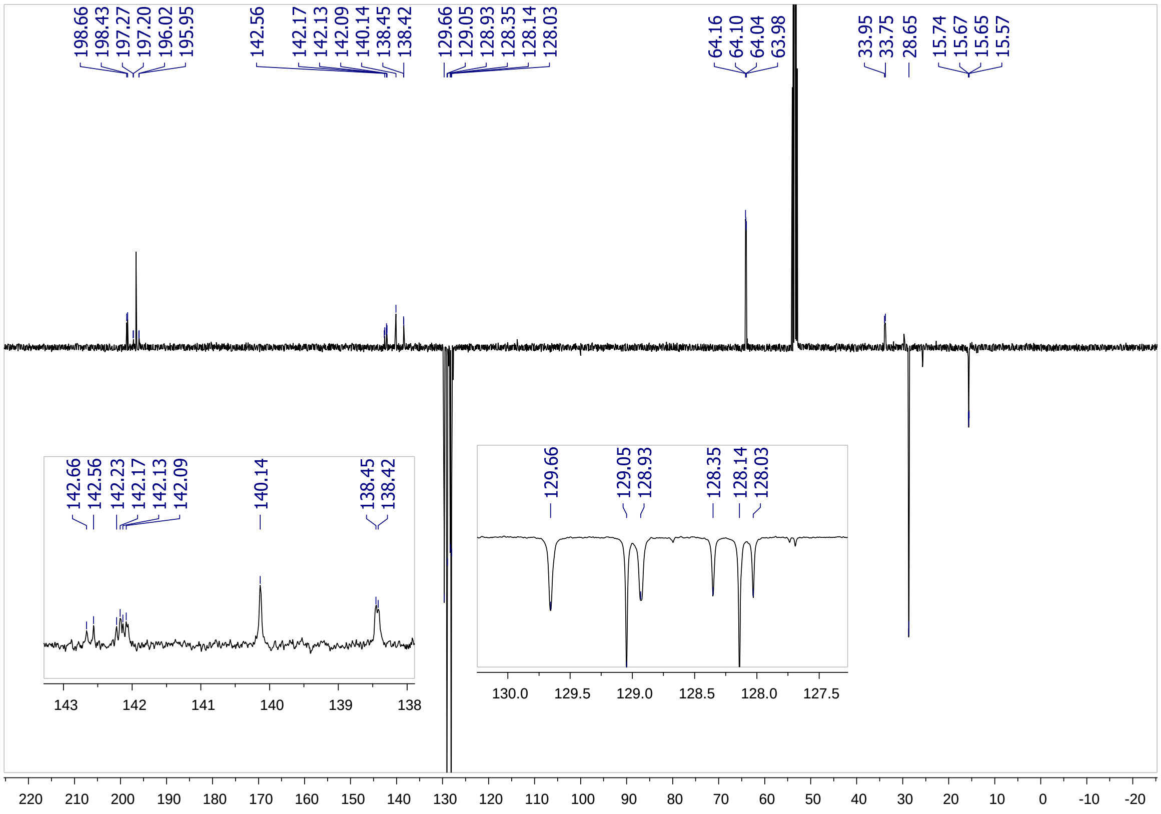
198.66 198.43 197.27 197.20 196.02 195.95
142.56 142.17 142.13 142.09 140.14 138.45 138.42
129.66 129.05 128.93 128.35 128.14 128.03
64.16 64.10 64.04 63.98
33.95 33.75 28.65
15.74 15.67 15.65 15.57
142.66 142.56 142.23 142.17 142.13 142.09 140.14 138.45 138.42
143 142 141 140 139 138
129.66 129.05 128.93 128.35 128.14 128.03
130.0 129.5 129.0 128.5 128.0 127.5
220 210 200 190 180 170 160 150 140 130 120 110 100 90 80 70 60 50 40 30 20 10 0 -10 -20

NMR data for complex 8a (^{1}H, ^{31}P, ^{13}C)

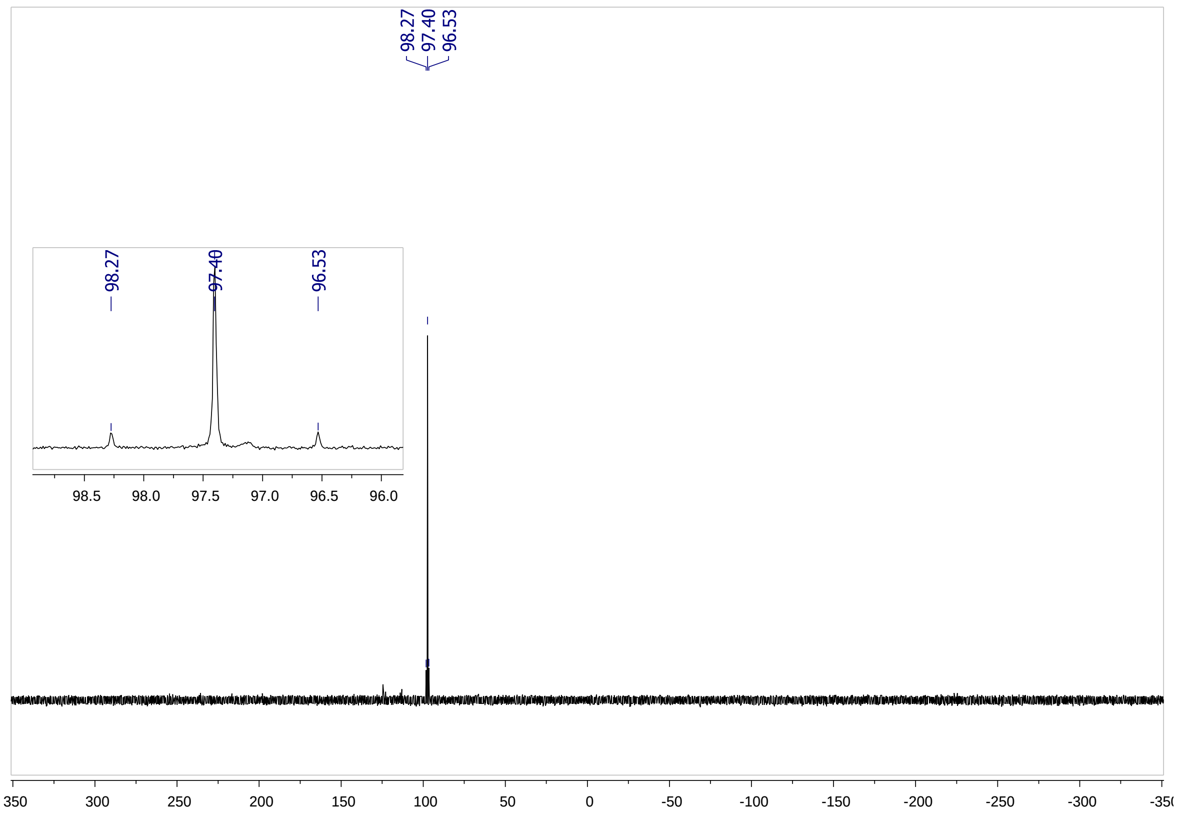

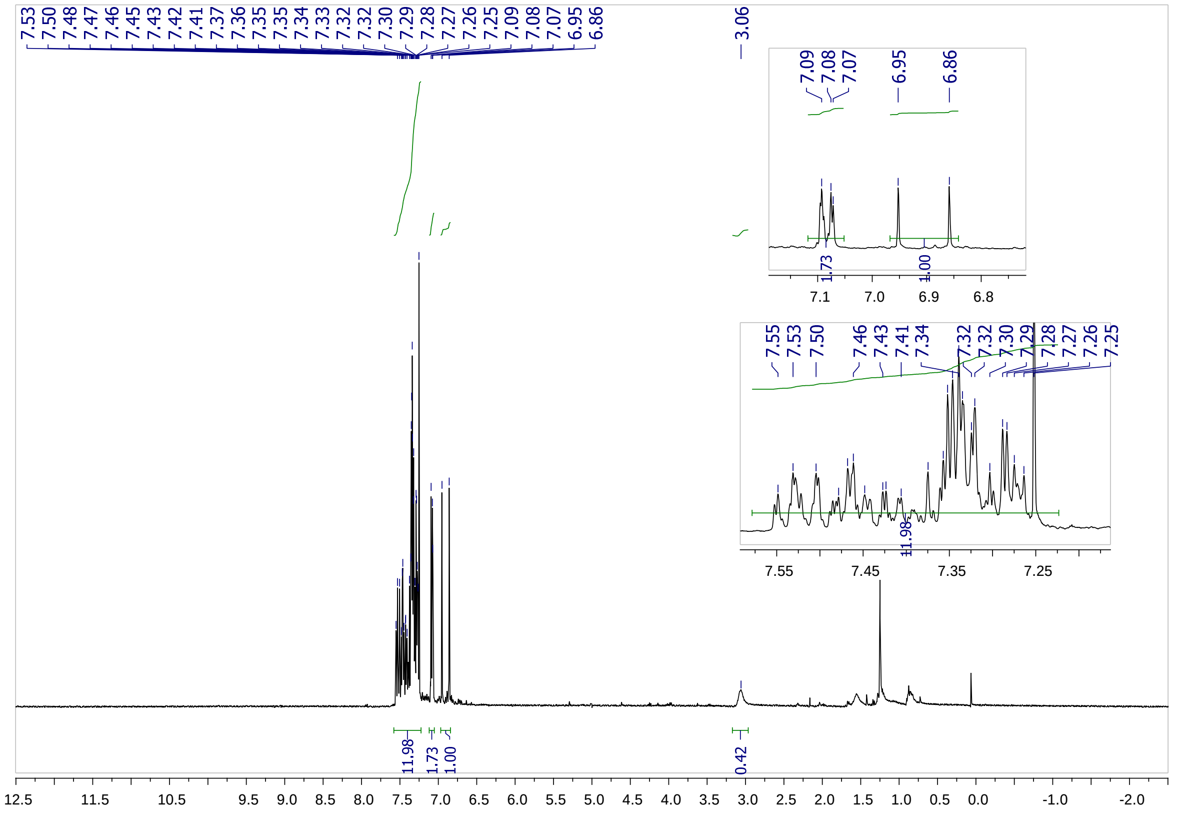

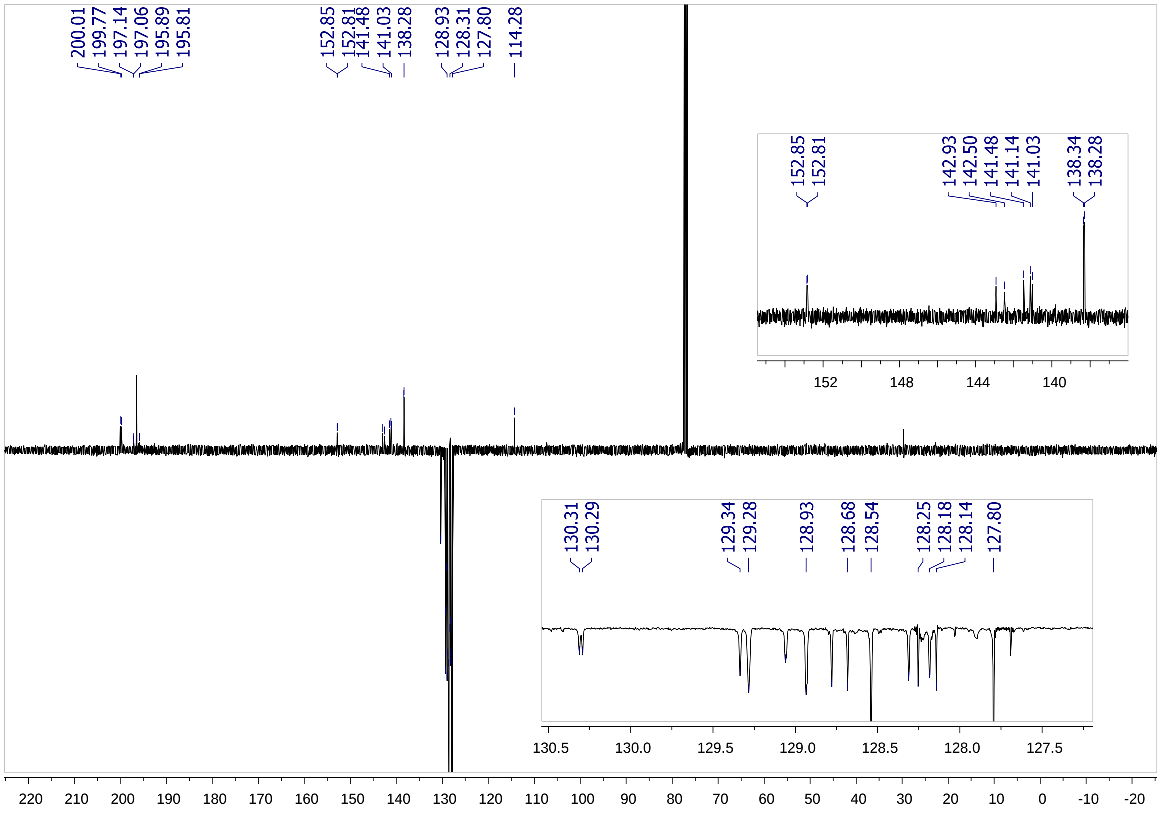

NMR data for complex 8b (^{1}H, ^{31}P, ^{13}C)

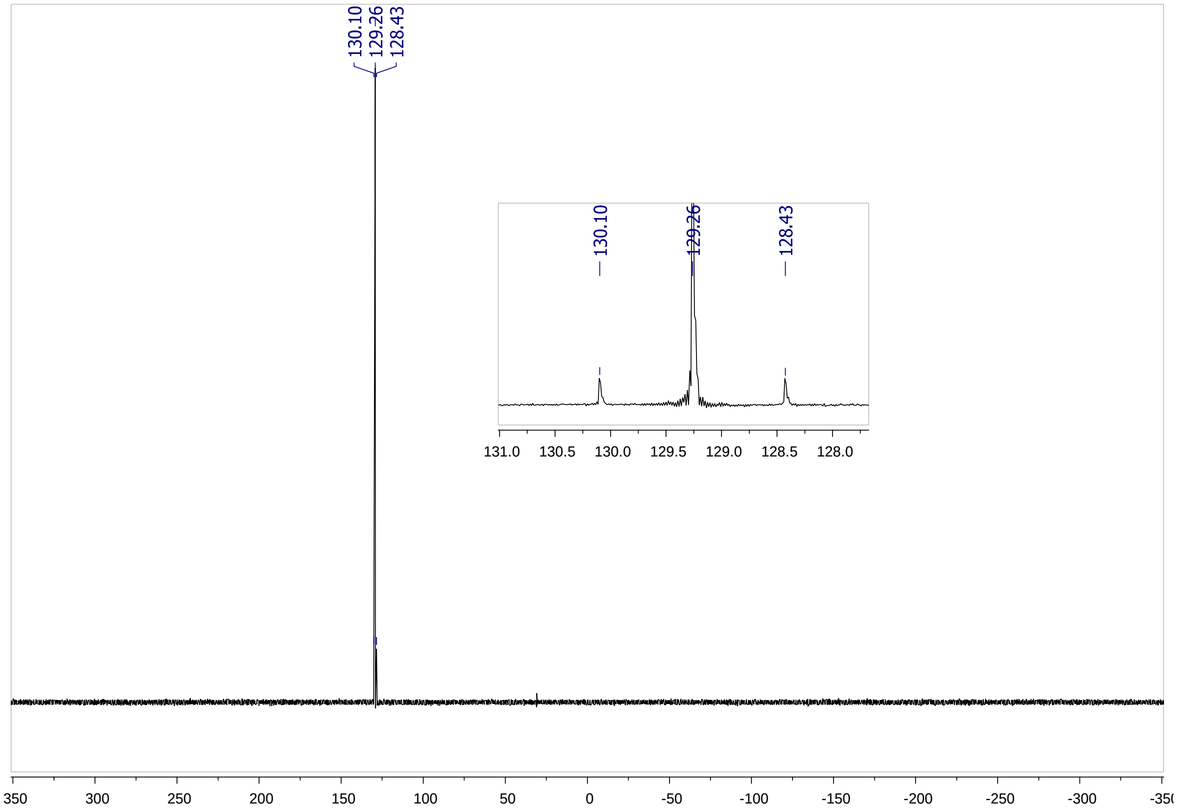

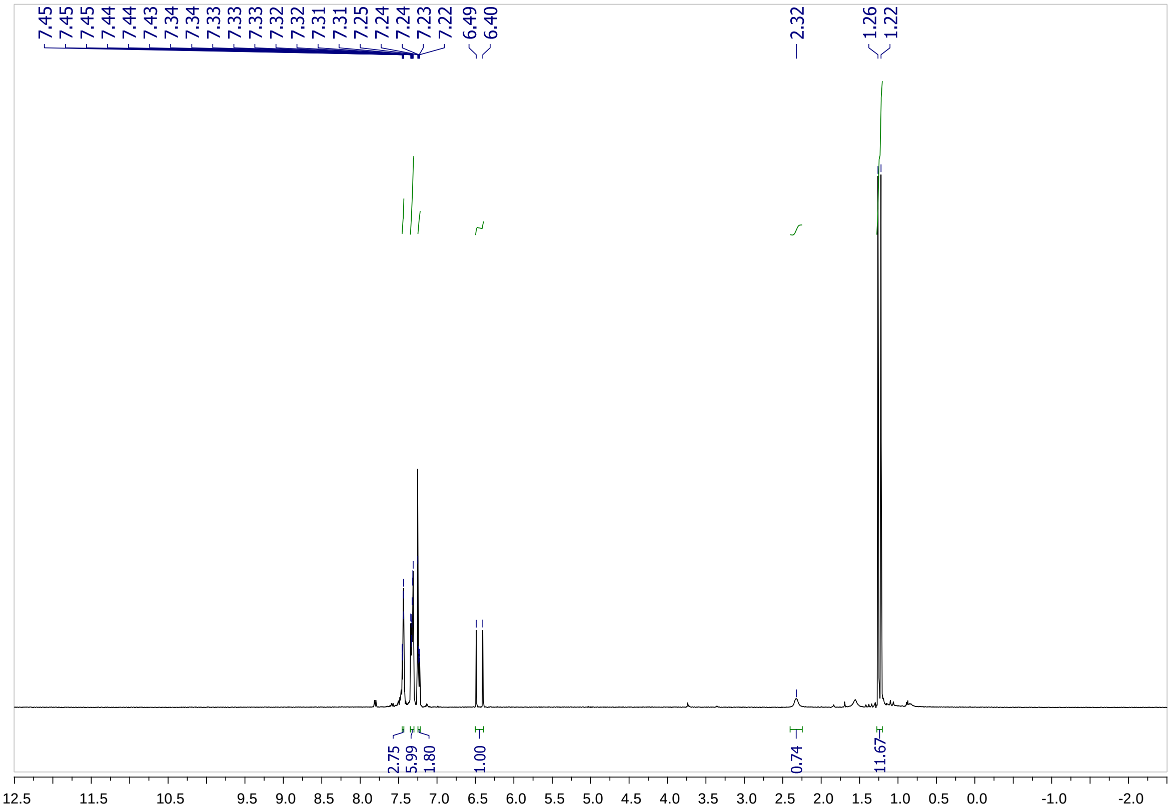
7.45
7.45
7.45
7.44
7.44
7.43
7.34
7.34
7.33
7.33
7.33
7.32
7.32
7.31
7.31
7.25
7.24
7.24
7.23
7.22
6.49
6.40
2.32
1.26
1.22
2.75
5.99
1.80
1.00
0.74
11.67

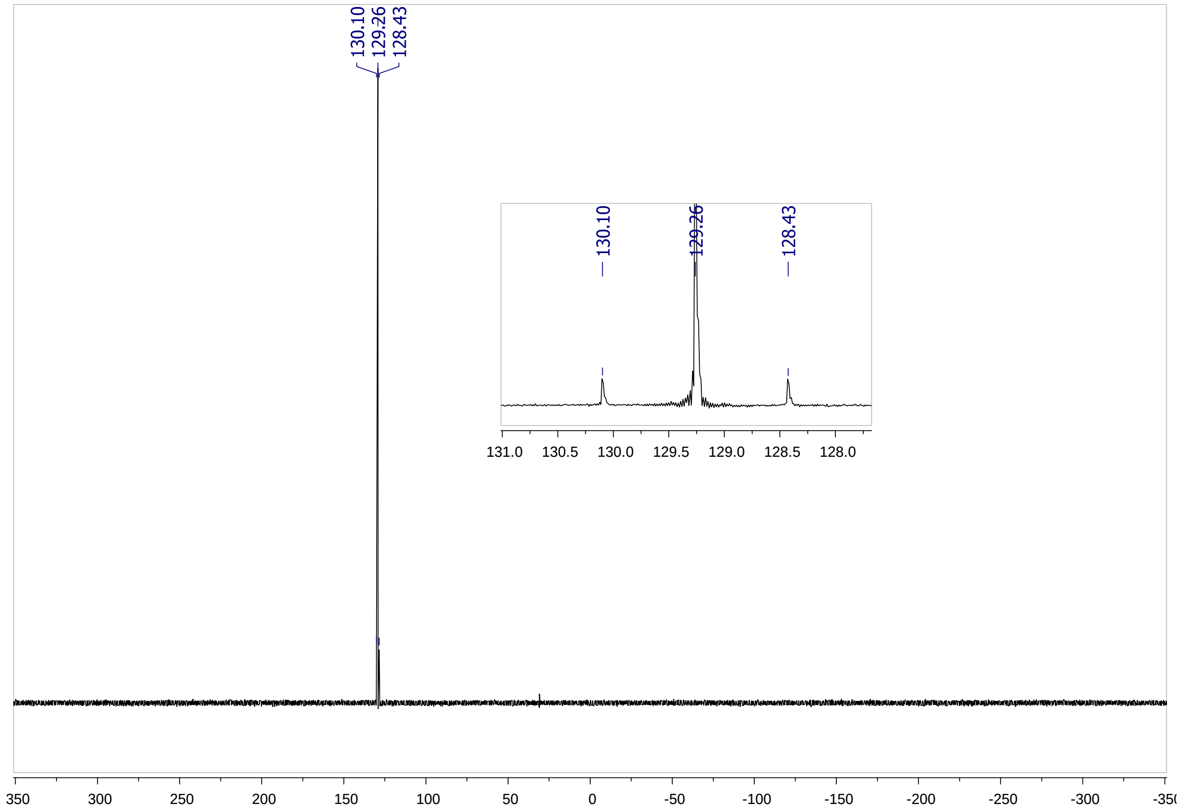
130.10
129.26
128.43

NMR data for complex 8c (^1H, ^{31}P, ^{13}C)

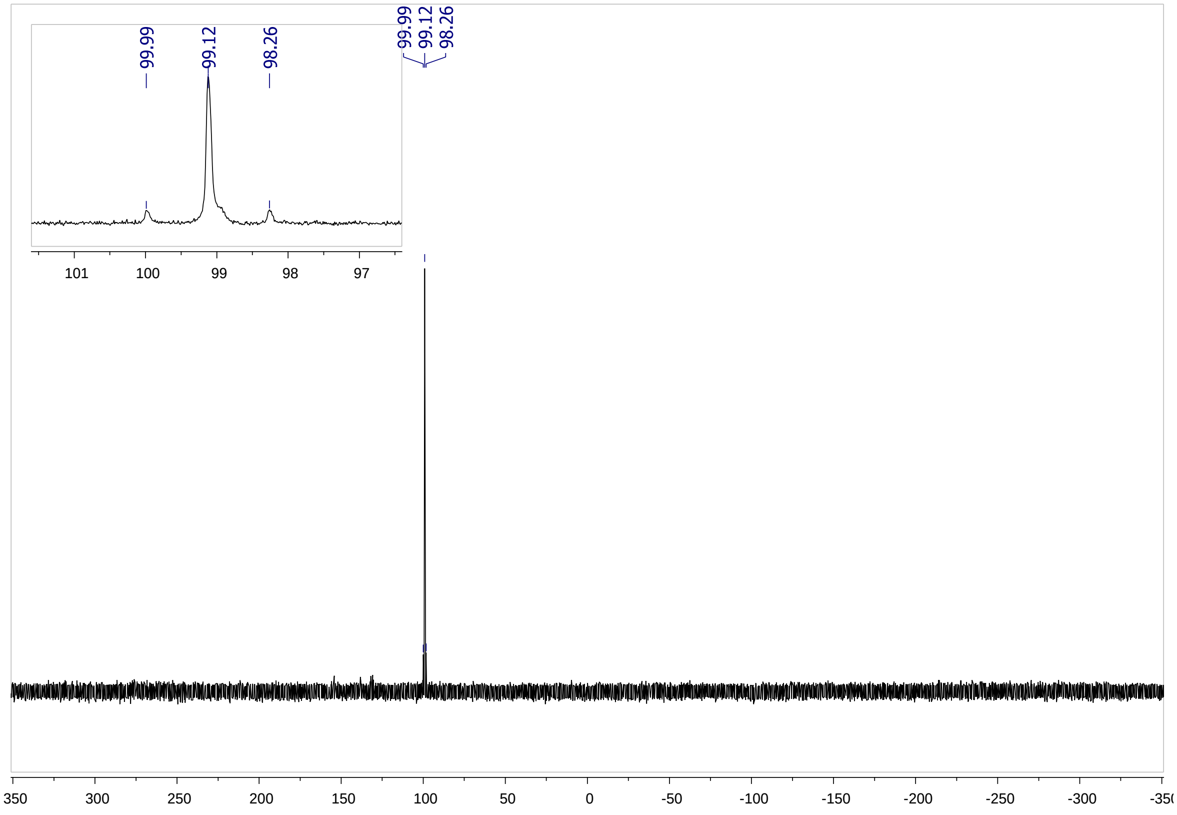

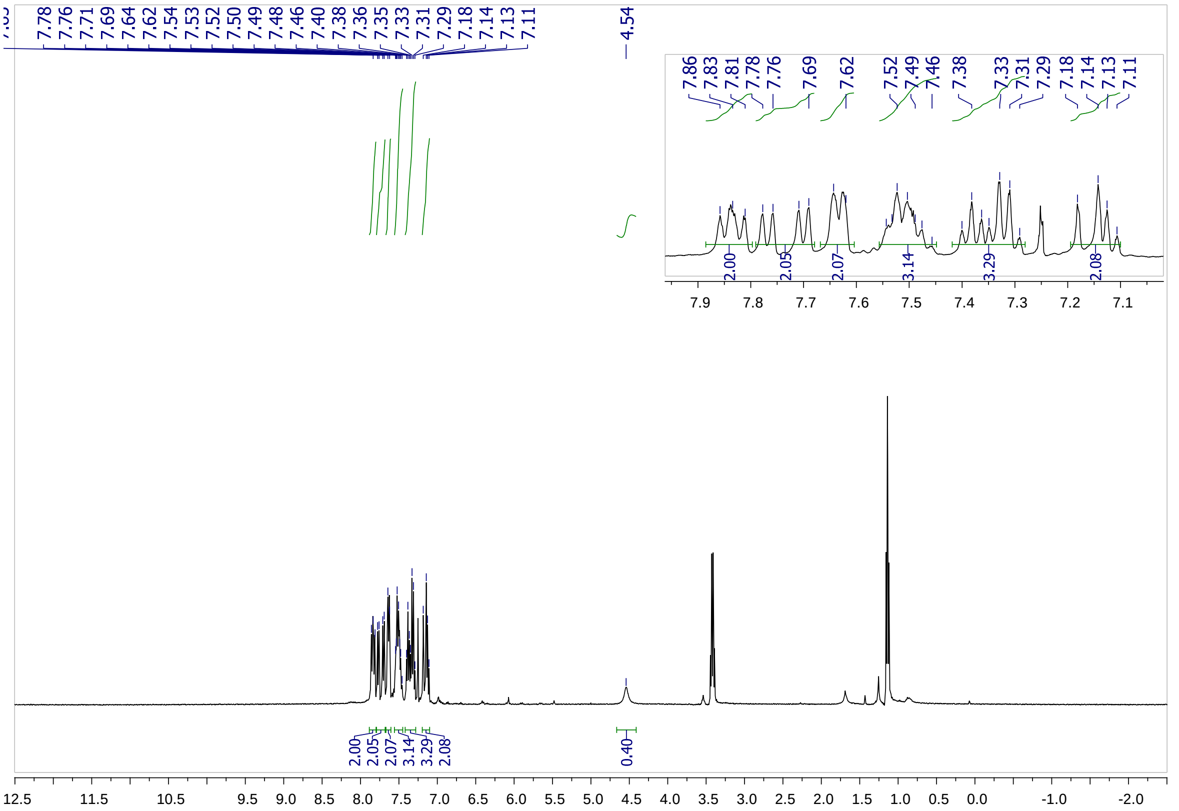

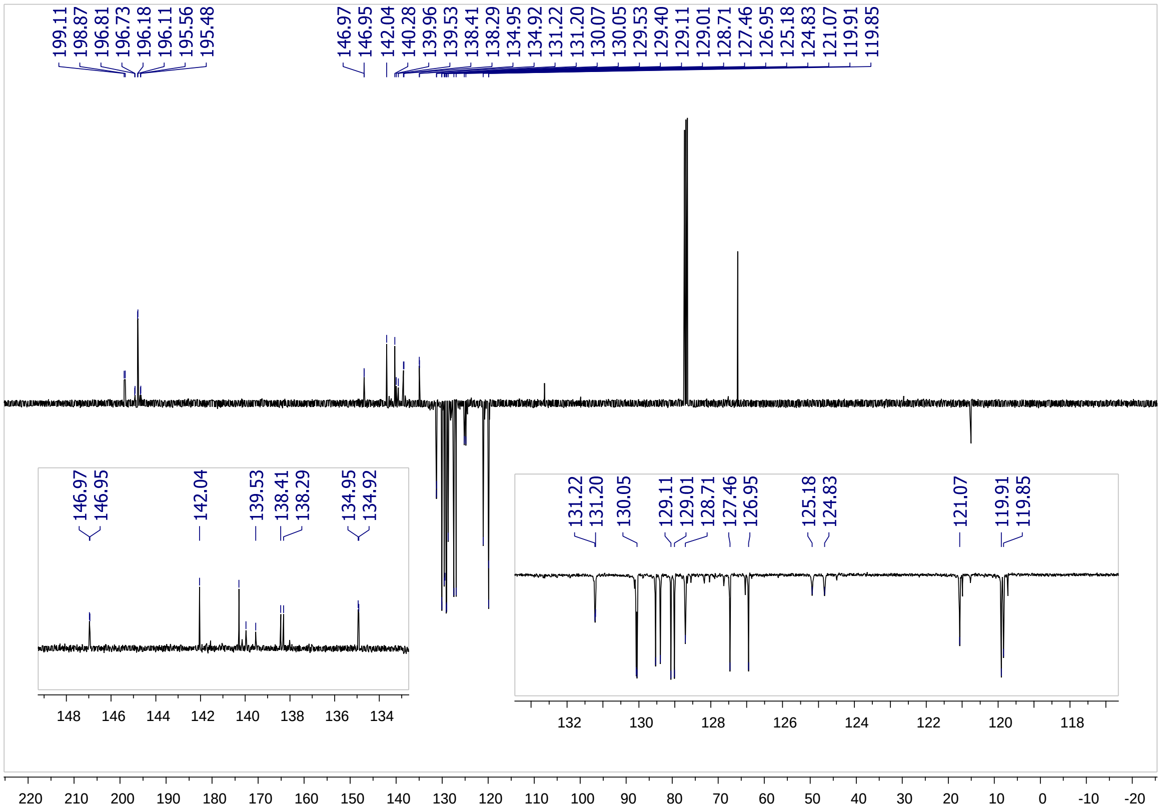

NMR data for complex 8d (^1H, ^{31}P, ^{13}C)

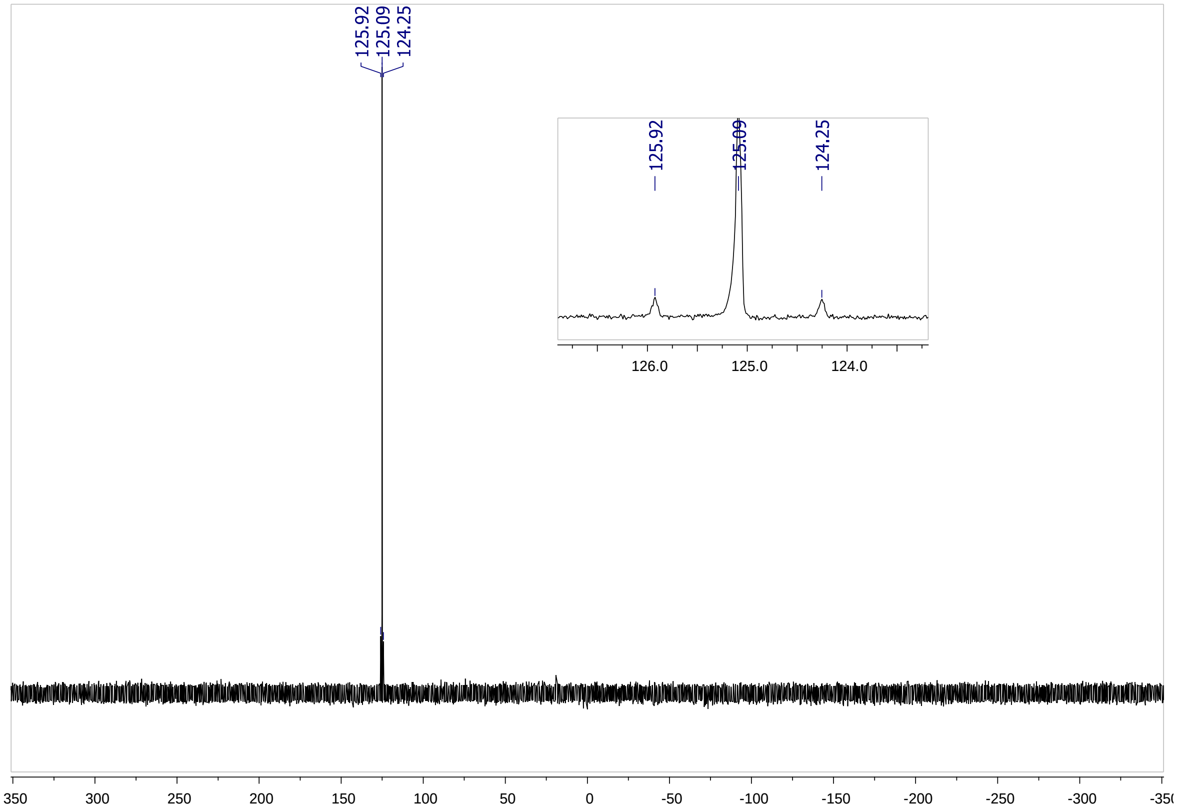

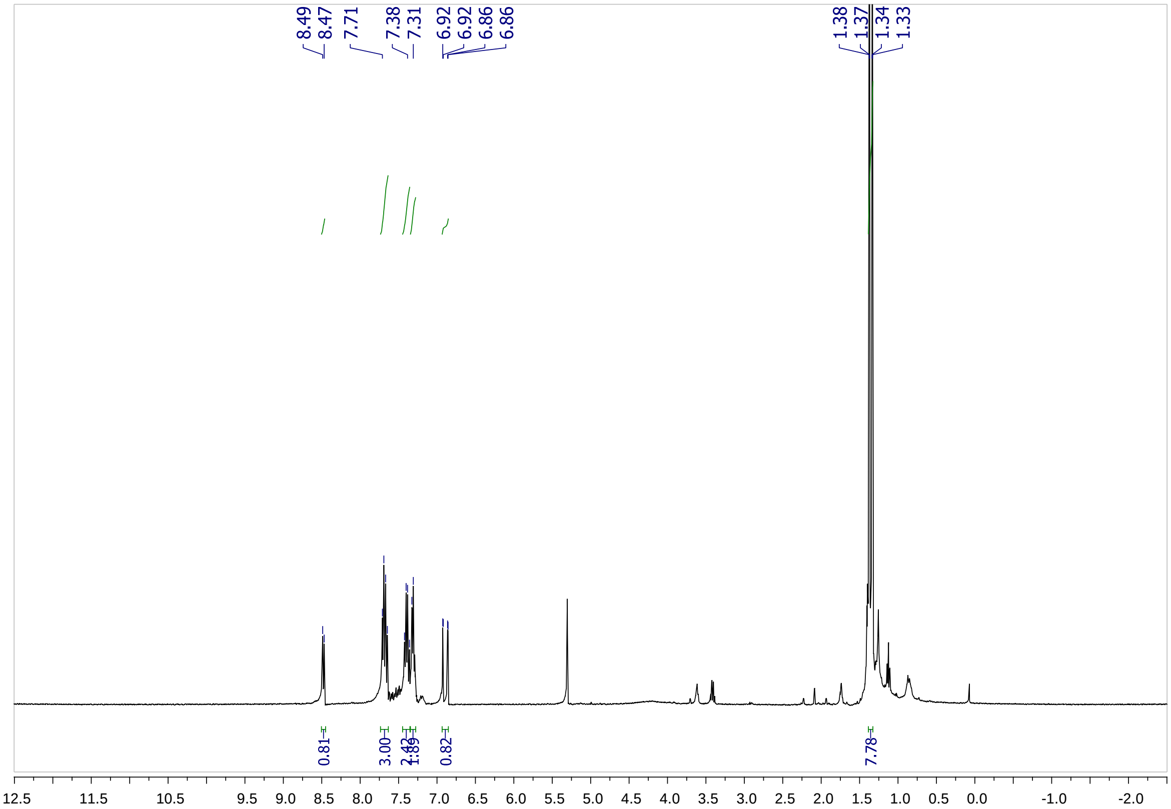
8.49
8.47
7.71
7.38
7.31
6.92
6.92
6.86
6.86
1.38
1.37
1.34
1.33
0.81
3.00
2.42
1.89
0.82
7.78

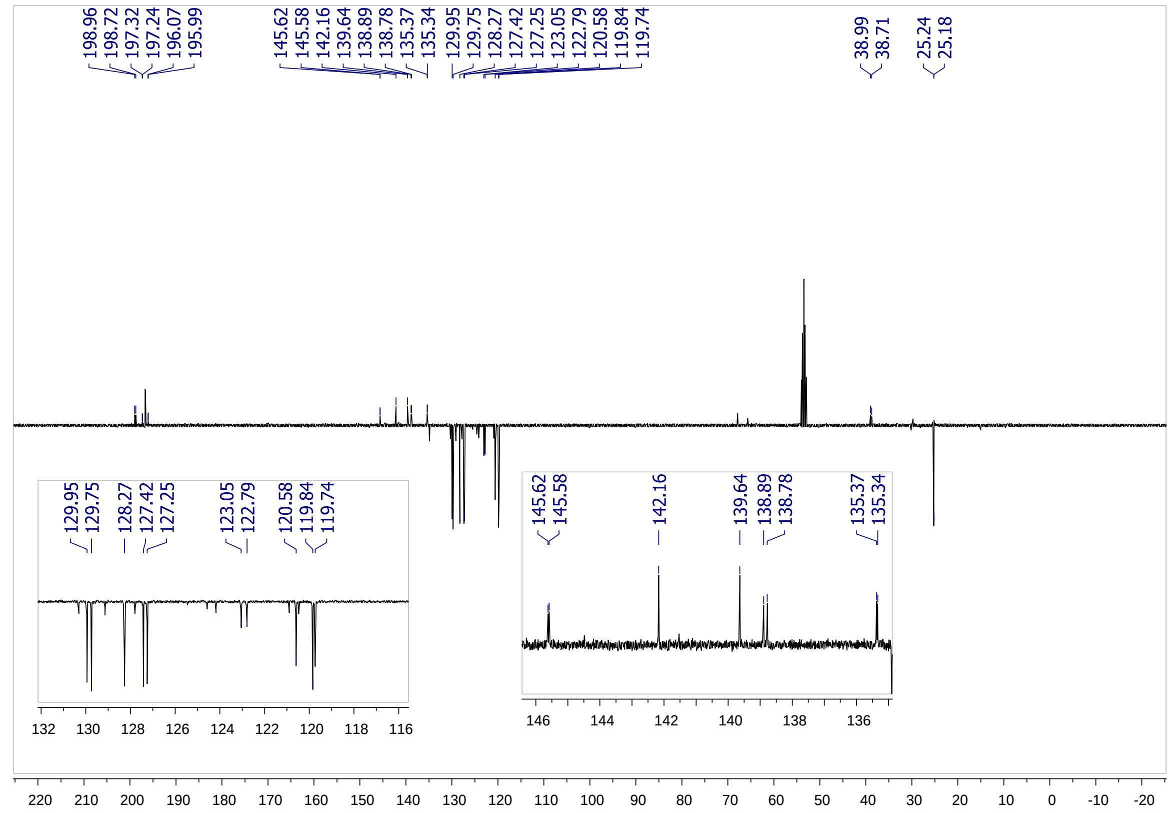
198.96
198.72
197.32
197.24
196.07
195.99
145.62
145.58
142.16
139.64
138.89
138.78
135.37
135.34
129.95
129.75
128.27
127.42
127.25
123.05
122.79
120.58
119.84
119.74
38.99
38.71
25.24
25.18

NMR data for complex 9a (^{1}H, ^{31}P, ^{13}C)

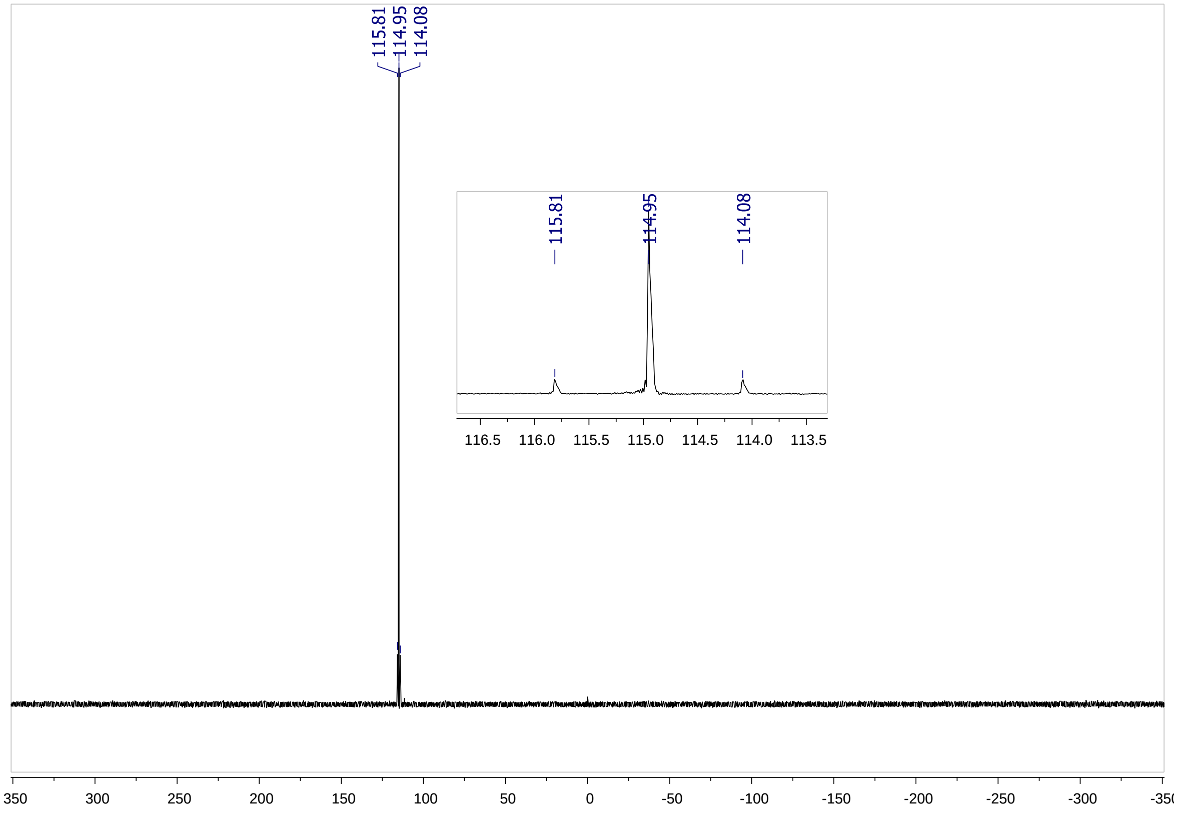

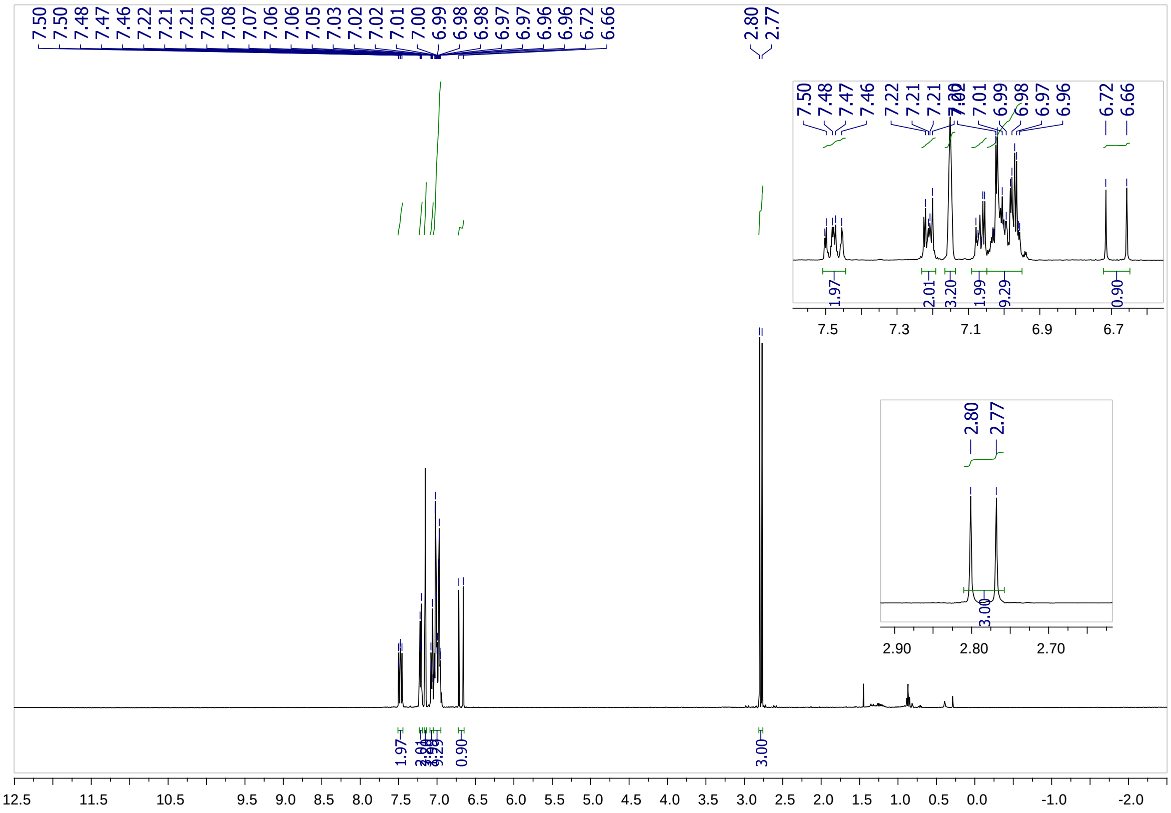

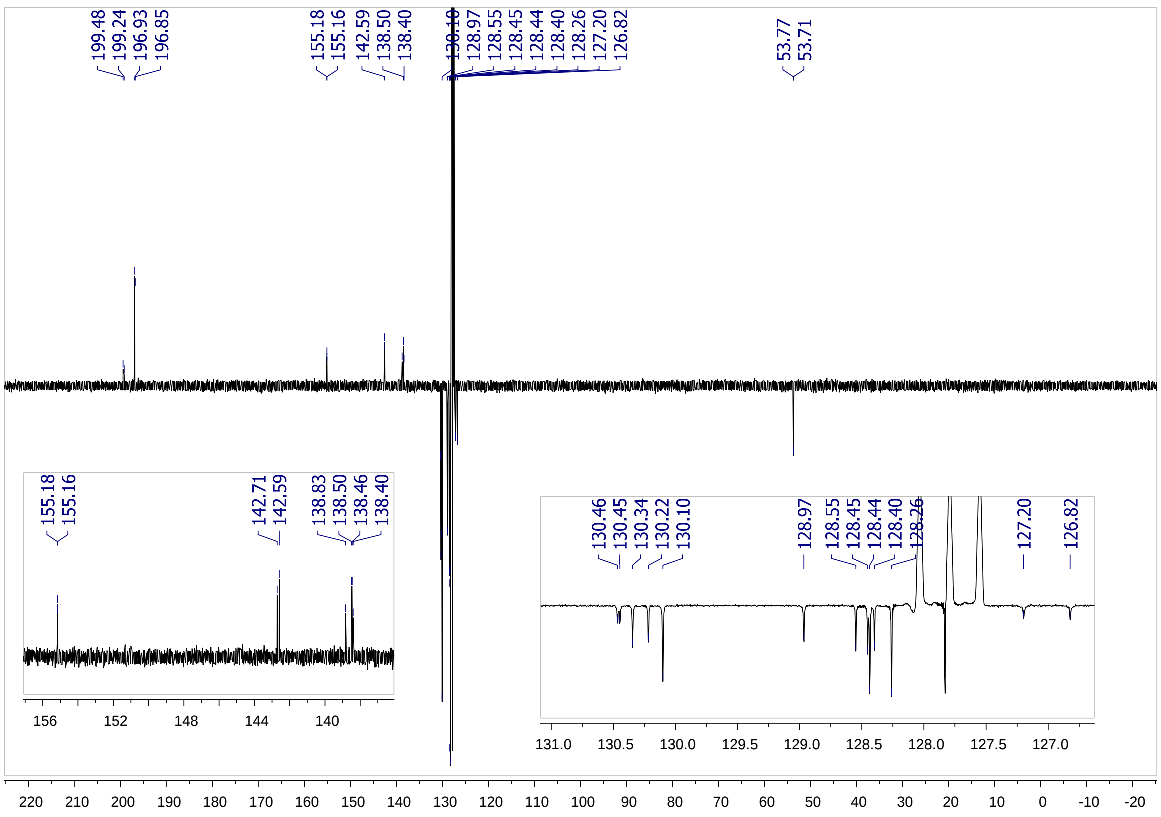

NMR data for complex 9b (^{1}H, ^{31}P, ^{13}C)

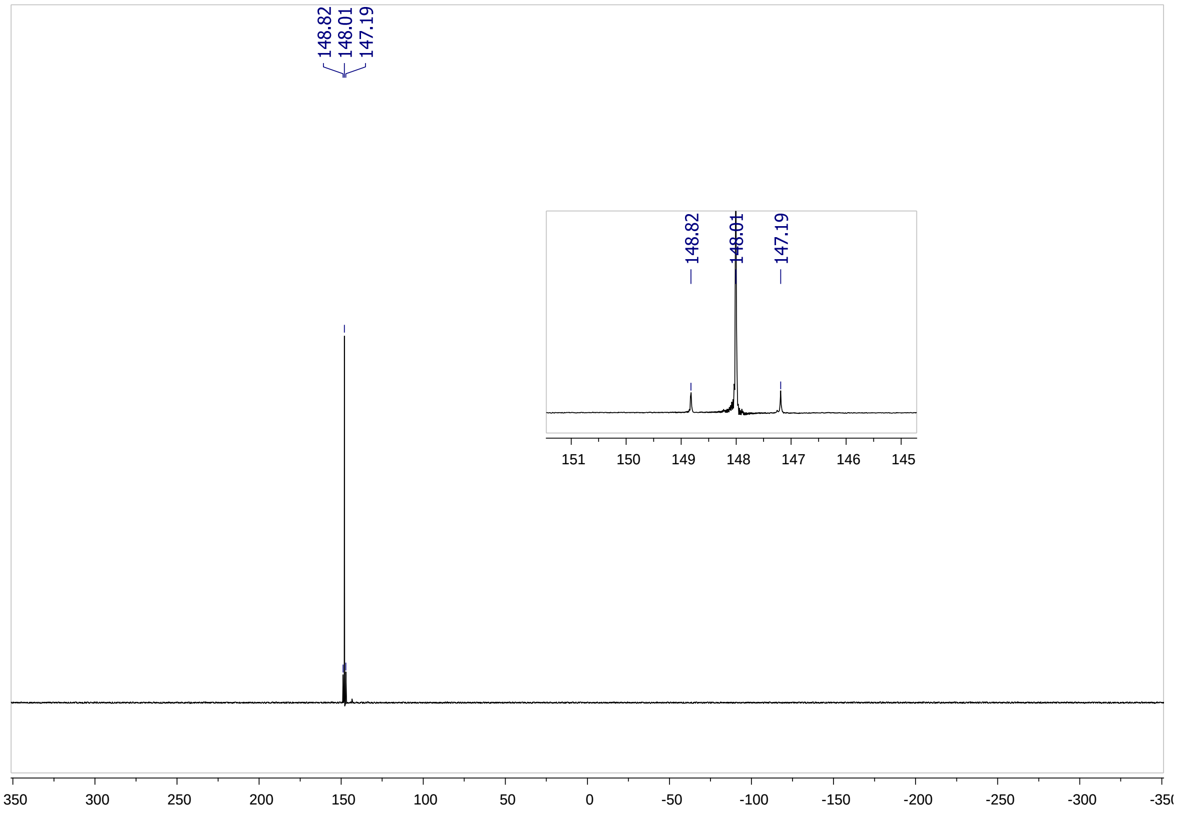

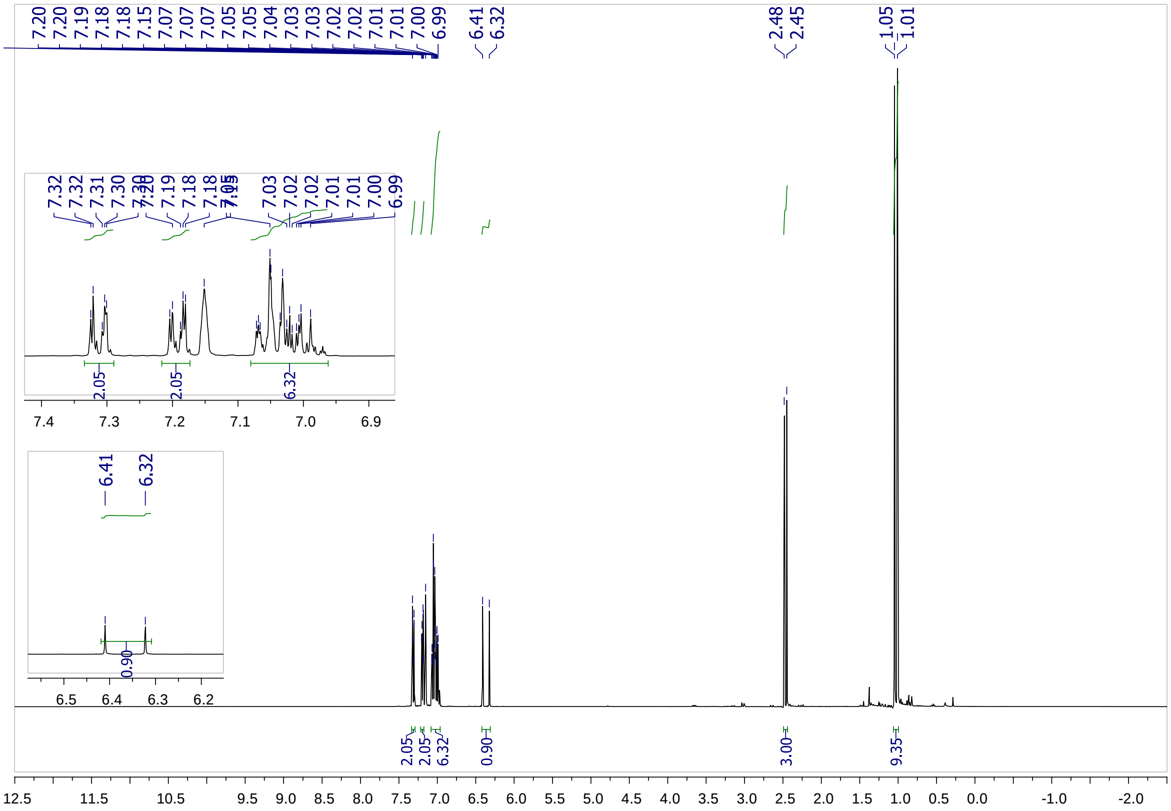
7.20 7.20 7.19 7.18 7.18 7.15 7.07 7.07 7.07 7.05 7.05 7.04 7.03 7.03 7.02 7.02 7.01 7.01 7.00 6.99
6.41 6.32
2.48 2.45
1.05 1.01
7.32 7.32 7.31 7.30 7.20 7.20 7.19 7.18 7.18 7.05 7.05 7.03 7.02 7.02 7.01 7.01 7.00 6.99
2.05 2.05 6.32
7.4 7.3 7.2 7.1 7.0 6.9
6.41 6.32
0.90
6.5 6.4 6.3 6.2
2.05 2.05 6.32 0.90 3.00 9.35
12.5 11.5 10.5 9.5 9.0 8.5 8.0 7.5 7.0 6.5 6.0 5.5 5.0 4.5 4.0 3.5 3.0 2.5 2.0 1.5 1.0 0.5 0.0 -1.0 -2.0

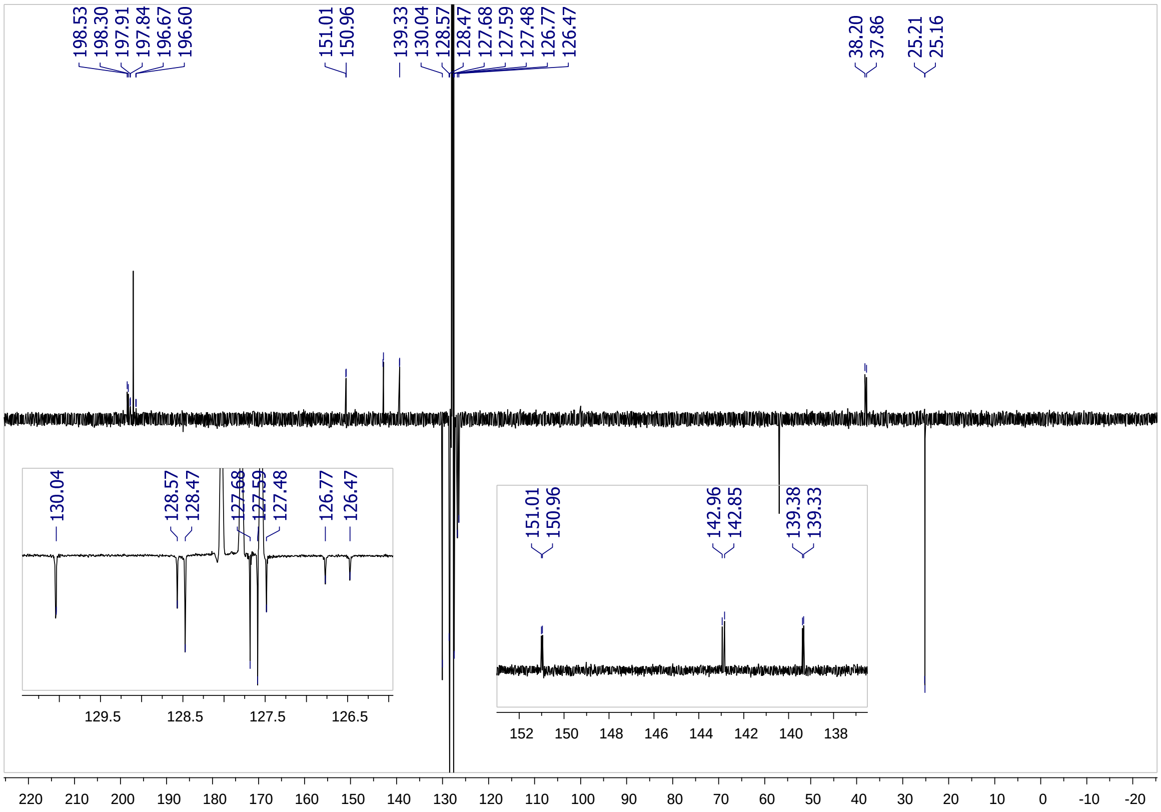
198.53 198.30 197.91 197.84 196.67 196.60
151.01 150.96
139.33 130.04 128.57 128.47 127.68 127.59 127.48 126.77 126.47
38.20 37.86
25.21 25.16
130.04 128.57 128.47 127.68 127.59 127.48 126.77 126.47
129.5 128.5 127.5 126.5
151.01 150.96 142.96 142.85 139.38 139.33
152 150 148 146 144 142 140 138
220 210 200 190 180 170 160 150 140 130 120 110 100 90 80 70 60 50 40 30 20 10 0 -10 -20

References

1. Goll, J.M. and E. Fillion, *Tuning the Reactivity of Palladium Carbenes Derived from Diphenylketene.* Organometallics, 2008. **27**(14): p. 3622-3625.
2. Stollé, R. and F. Wolf, *Über einige Abkömmlinge der Diphenylen-essigsäure und Bis-diphenylen-bernsteinsäure.* Berichte der deutschen chemischen Gesellschaft, 1913. **46**(2): p. 2248-2252.
